# Supplementary material for: Synthesis and Anticancer Evaluation of 4-Anilinoquinolinylchalcone Derivatives
Source: Int J Mol Sci. 2023 Mar 23;24(7):6034. doi: 10.3390/ijms24076034 (PMC10094048; doi:10.3390/ijms24076034)

YCY-4895

Pulse Sequence: s2pul

Mercury-400BB "MercuryPlus400"
Date: May 8 2014
Solvent: DMSO
Ambient temperature
Total 32 repetitions

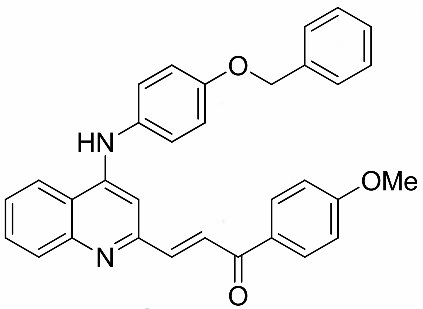

4a

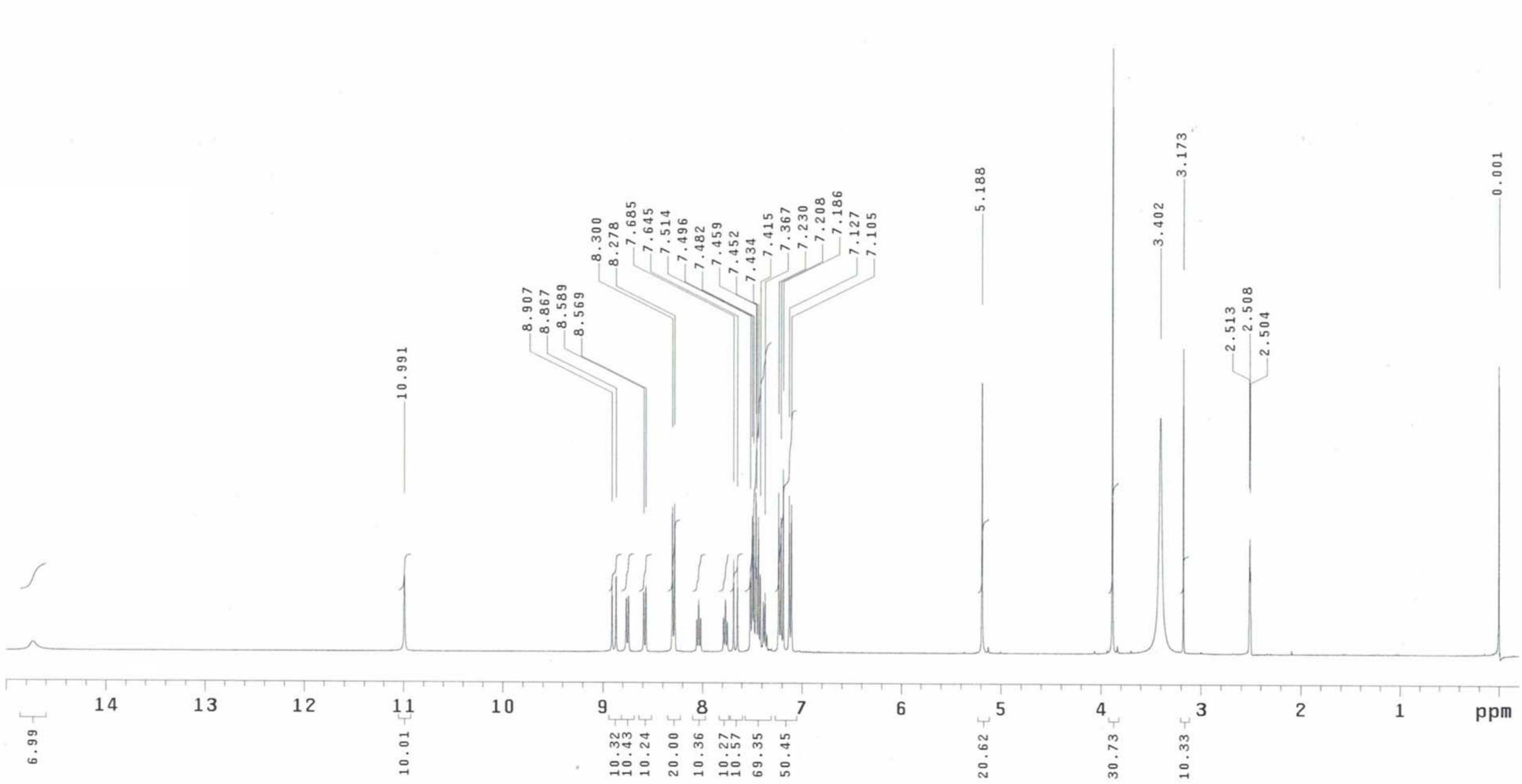

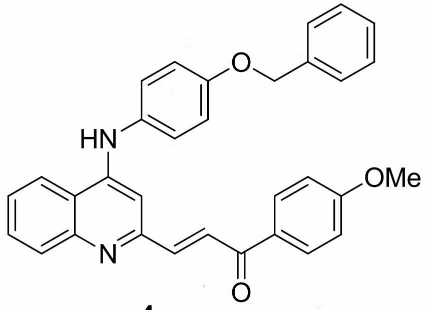
O
HN
OMe
N
O

4a

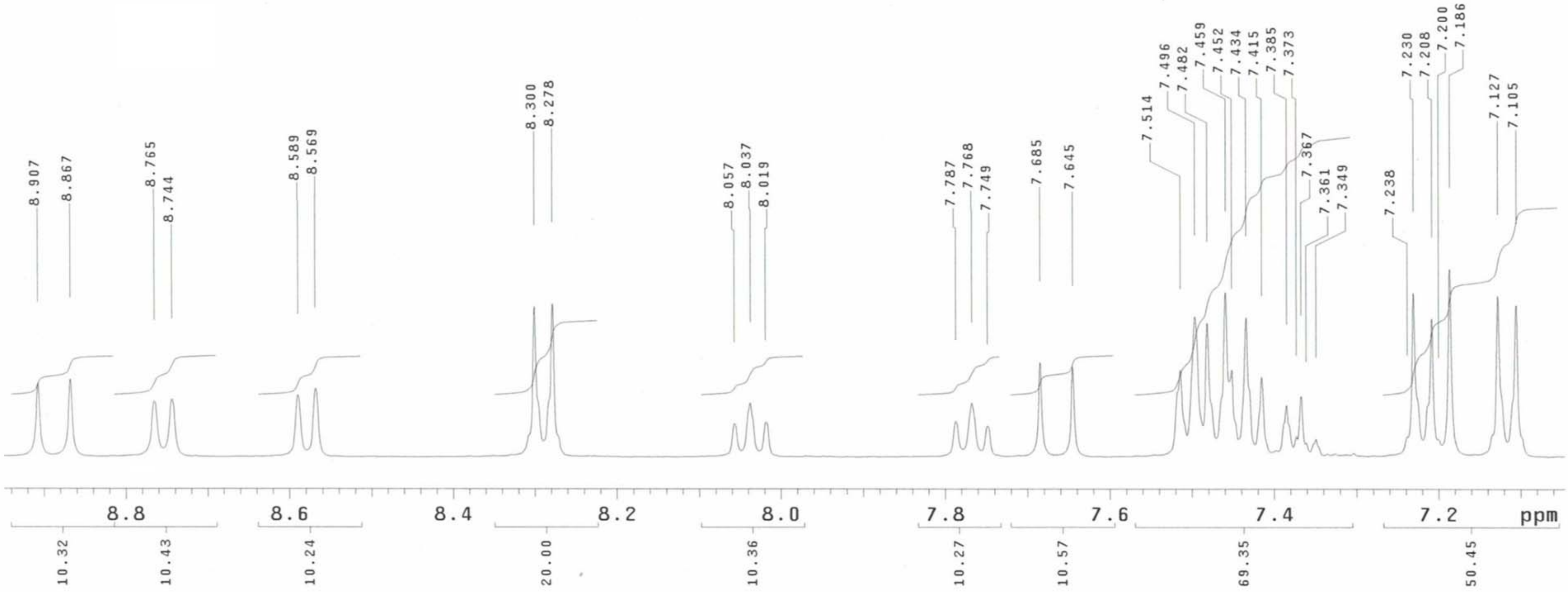
8.907
8.867
8.765
8.744
8.589
8.569
8.300
8.278
8.057
8.037
8.019
7.787
7.768
7.749
7.685
7.645
7.514
7.496
7.482
7.459
7.452
7.434
7.415
7.385
7.373
7.367
7.361
7.349
7.238
7.230
7.208
7.200
7.186
7.127
7.105
8.8
8.6
8.4
8.2
8.0
7.8
7.6
7.4
7.2
ppm
10.32
10.43
10.24
20.00
10.36
10.27
10.57
69.35
50.45

YCY-4895

Pulse Sequence: s2pul
Mercury-400BB "MerPlus400"
Date: Mar 13 2023
Solvent: dmso
Ambient temperature
Total 2208 repetitions

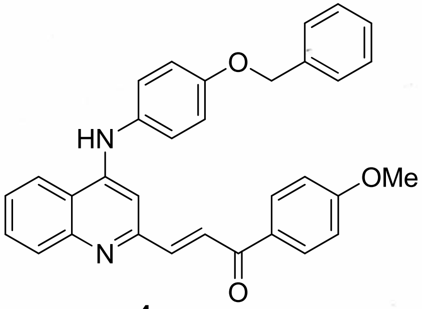

4a

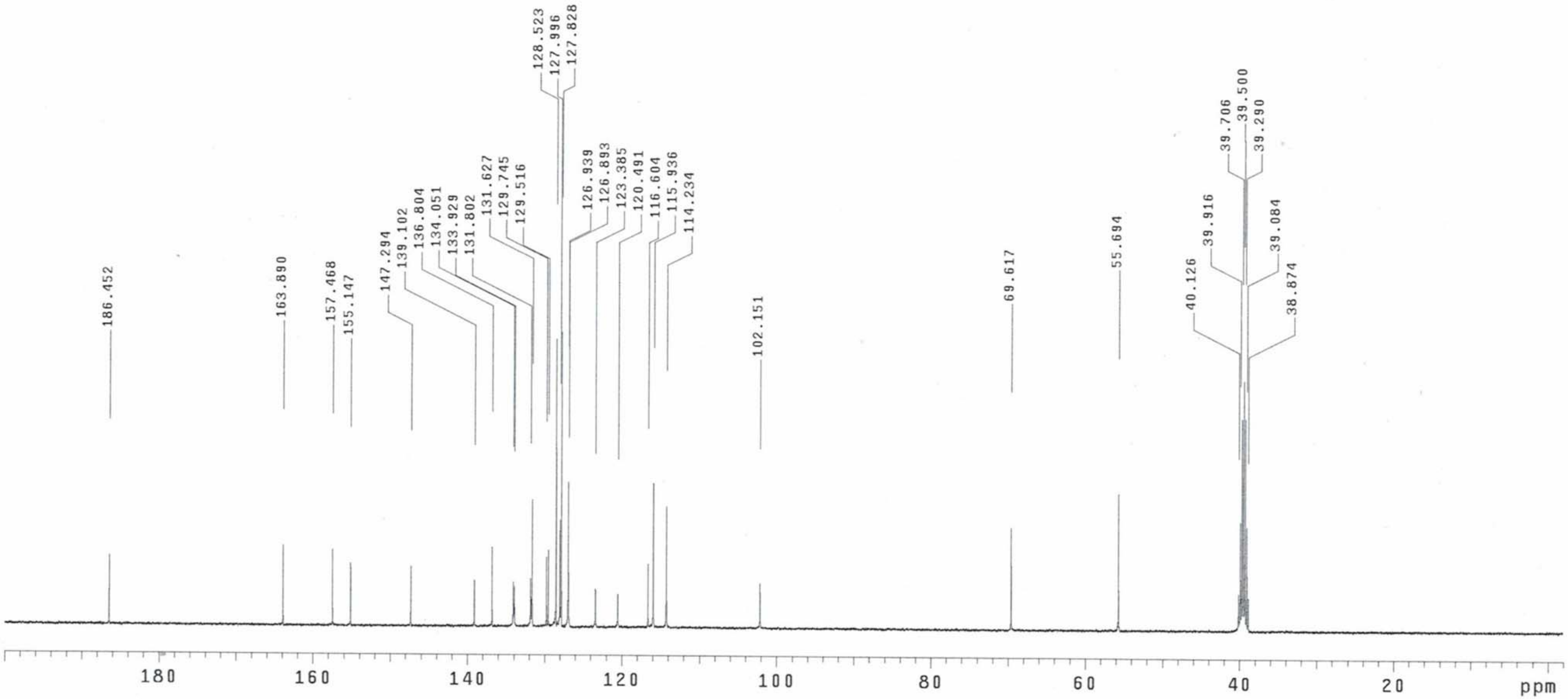

YCY-4895

Pulse Sequence: s2pul

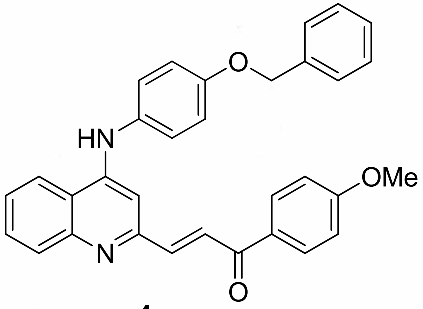

4a

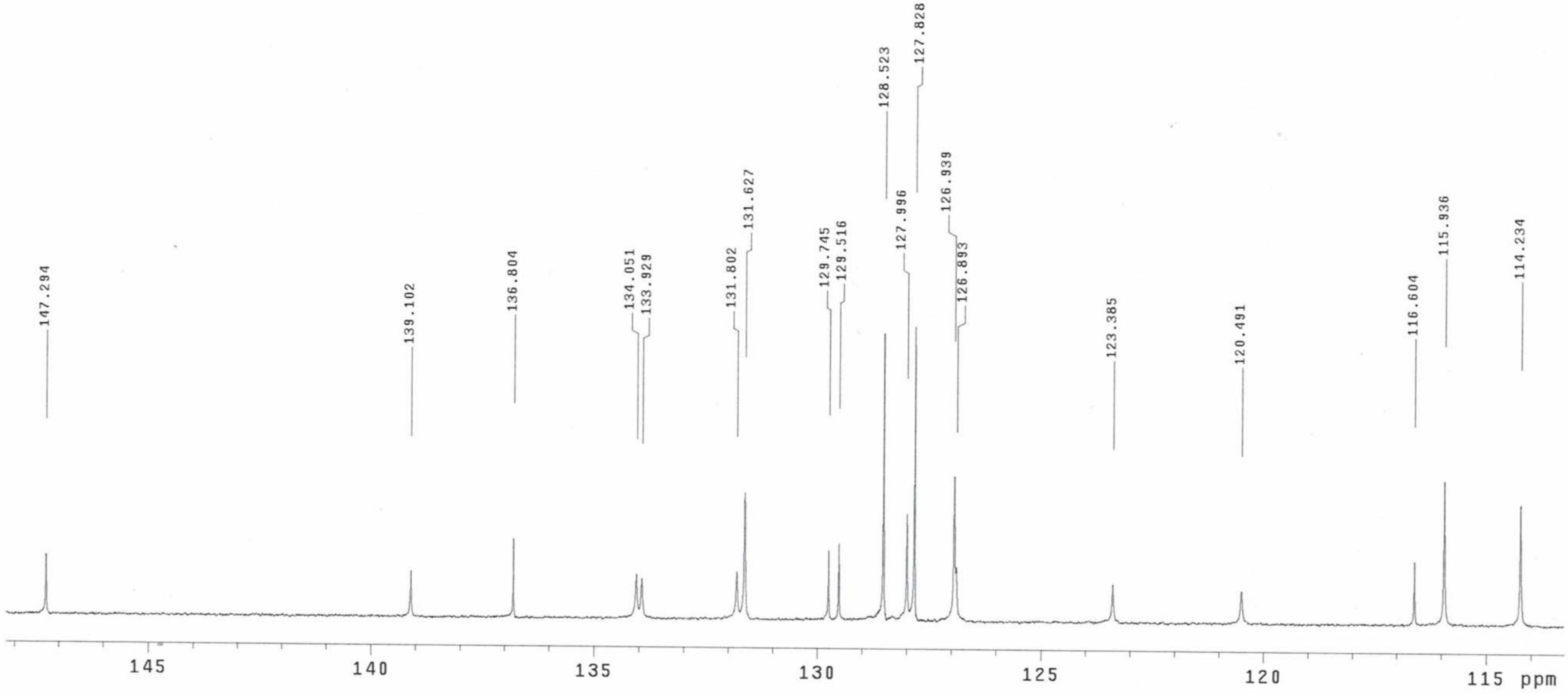

YCY-4895

Pulse Sequence: DEPT

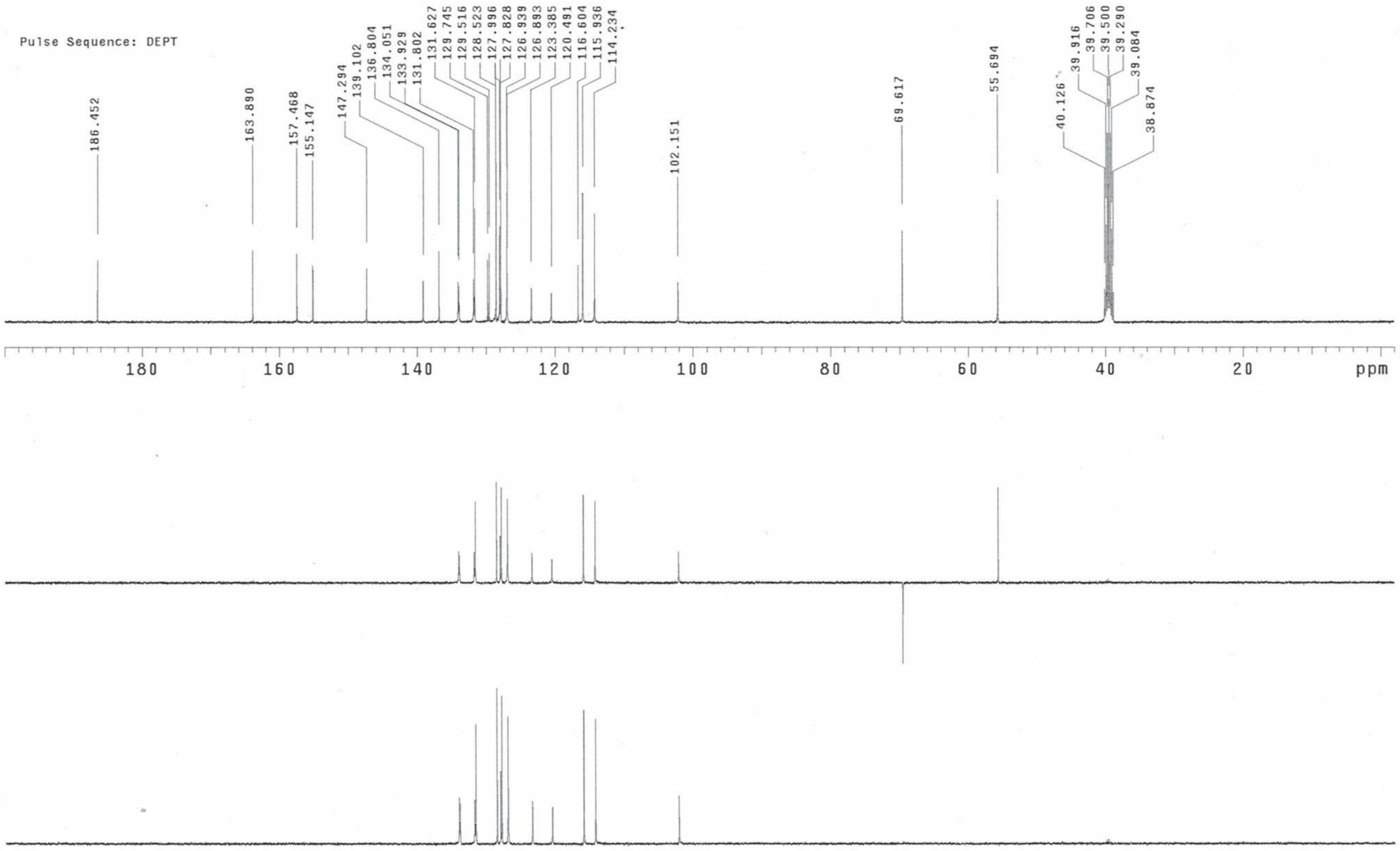

YCY-4895

Pulse Sequence: DEPT

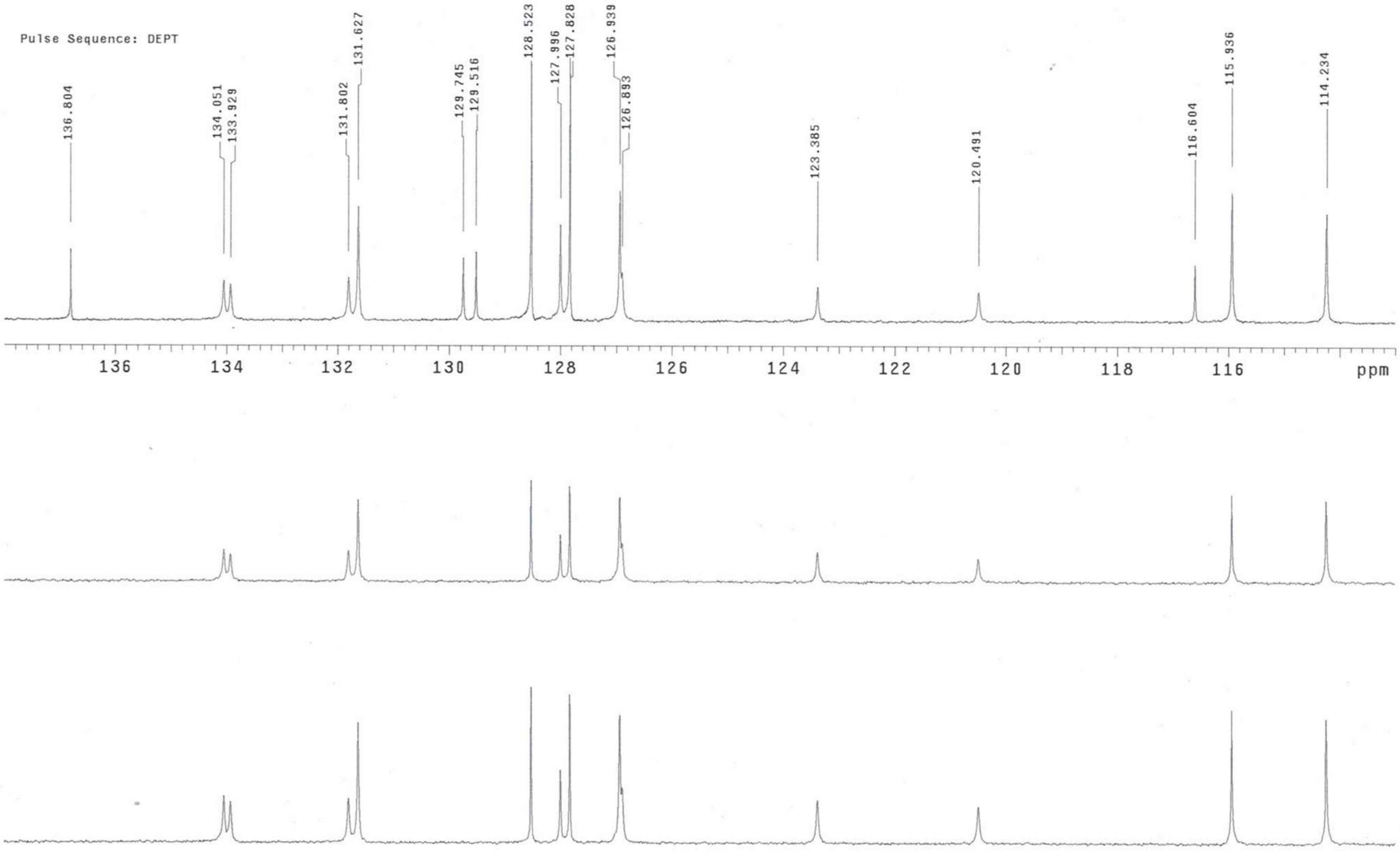

YCY-4900

Pulse Sequence: s2pul
UNITYplus-400 "unity400"
Date: Jun 6 2014
Solvent: DMSO
Ambient temperature
Total 48 repetitions

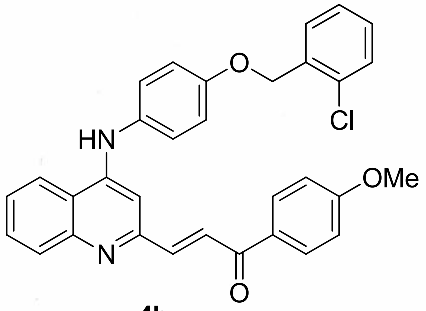

4b

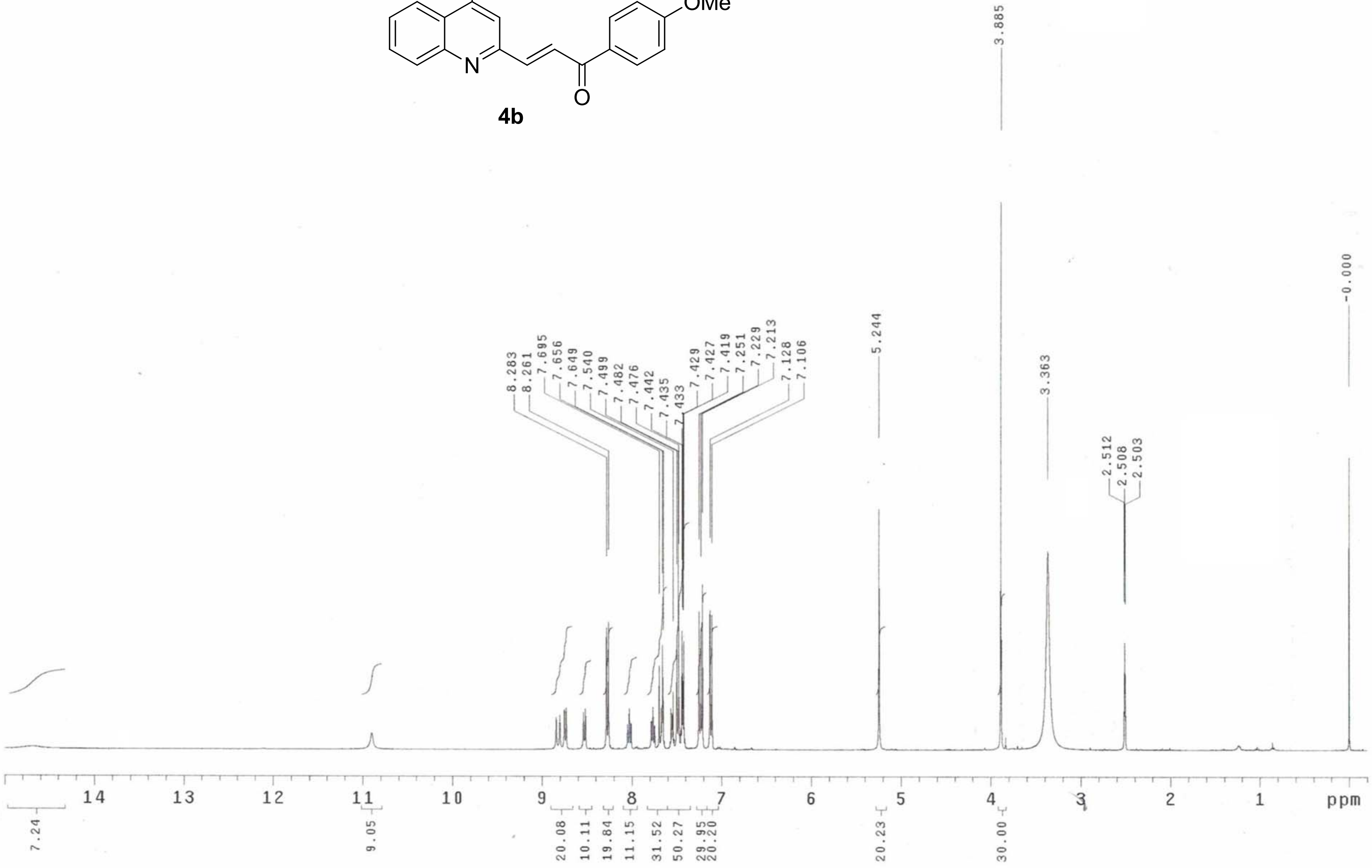

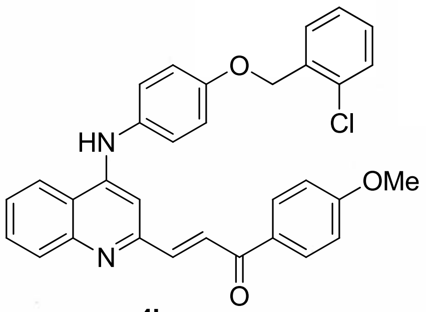

4b

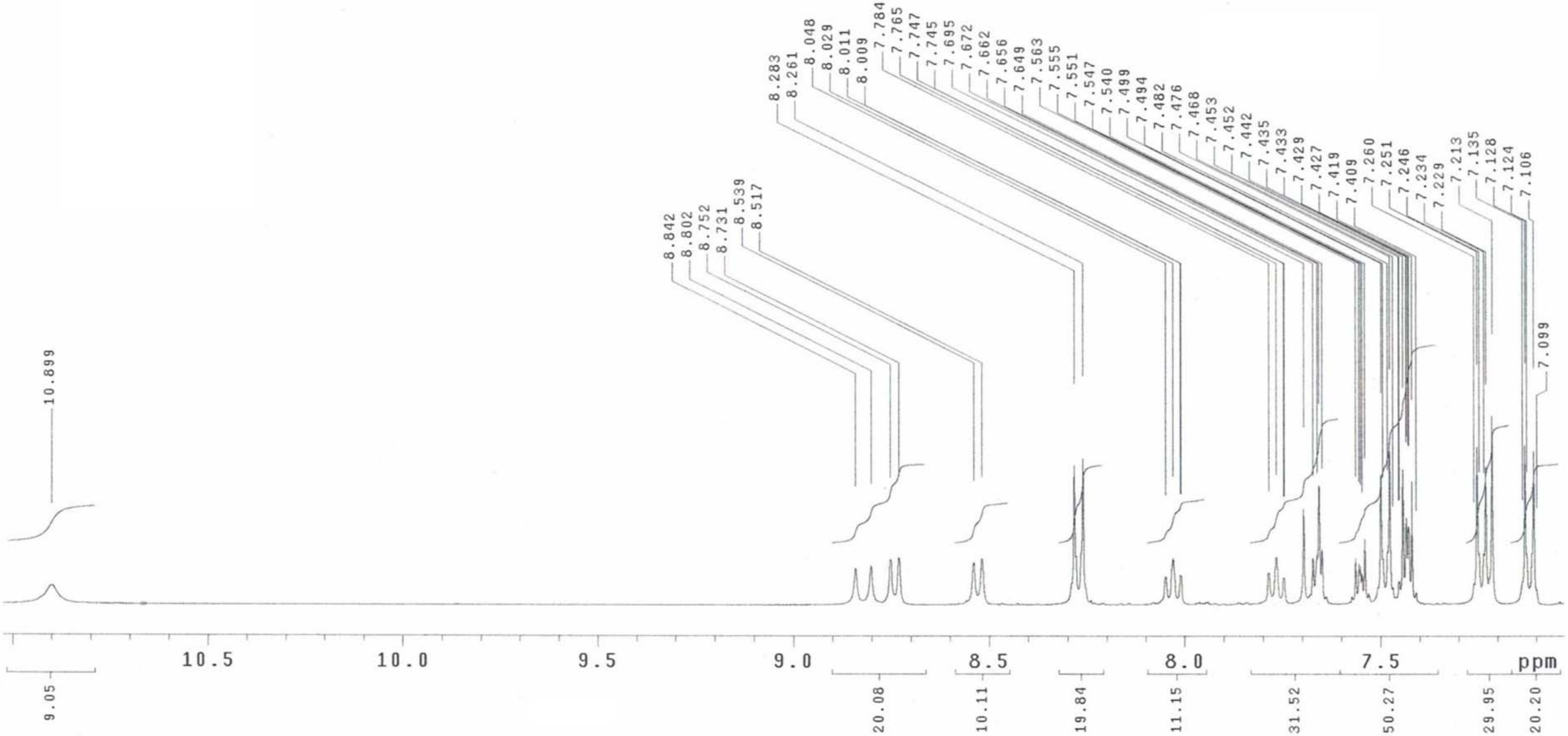

YCY-4900

Pulse Sequence: s2pul

UNITYplus-400 "unity400"
Date: Jun 6 2014
Solvent: DMSO
Ambient temperature
Total 33456 repetitions

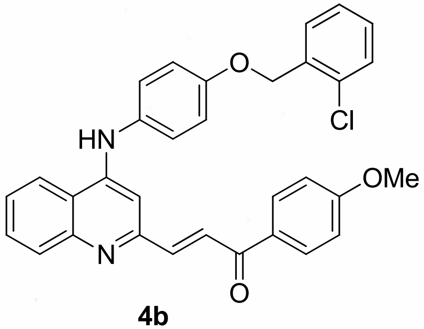

4b

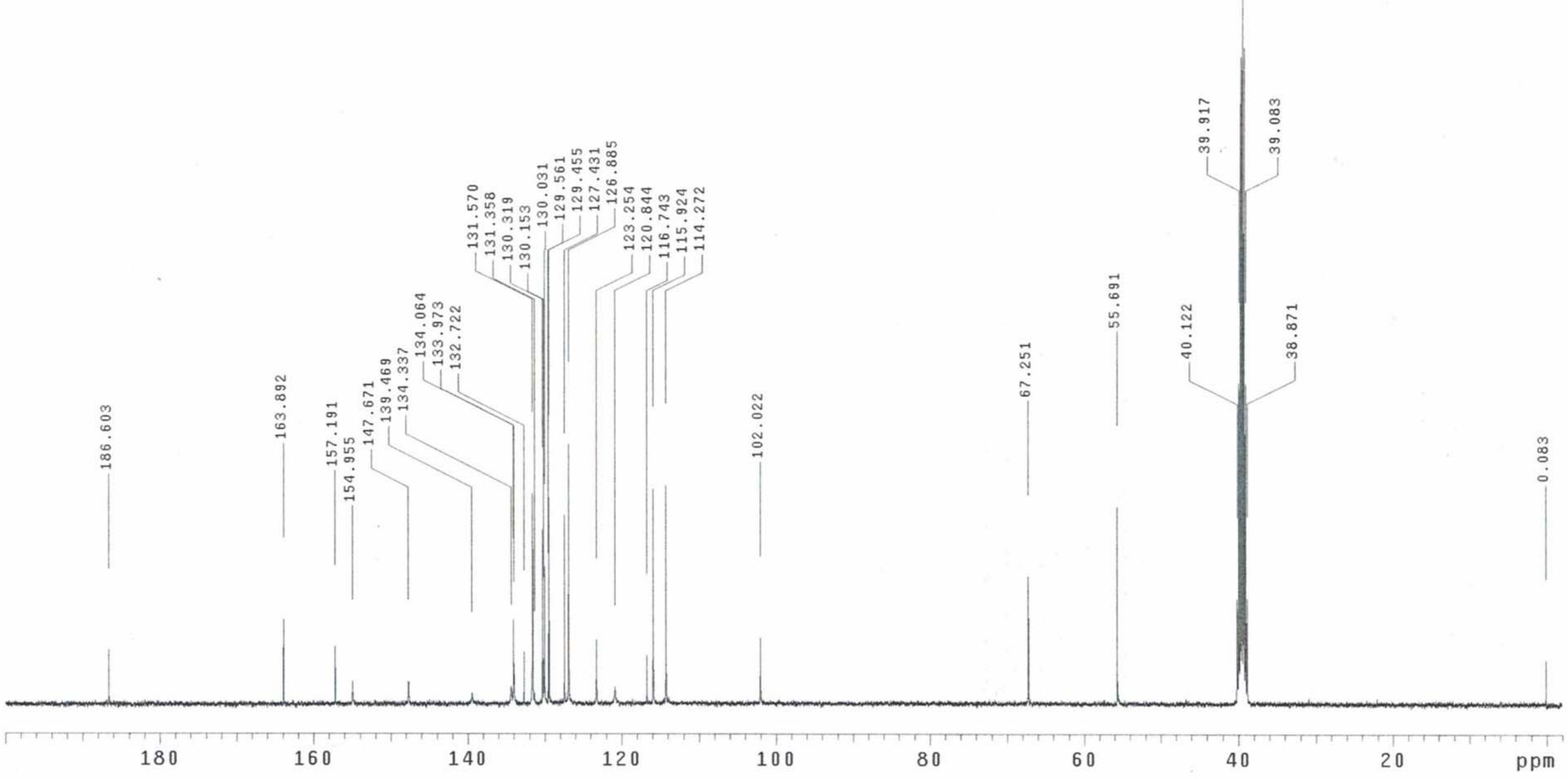

YCY-5458

Pulse Sequence: s2pul
Mercury-400BB "MercuryPlus400"
Date: Sep 3 2014
Solvent: DMSO
Ambient temperature
Total 64 repetitions

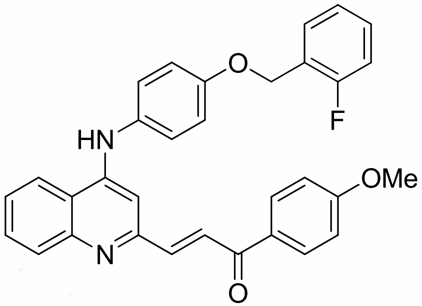

4c

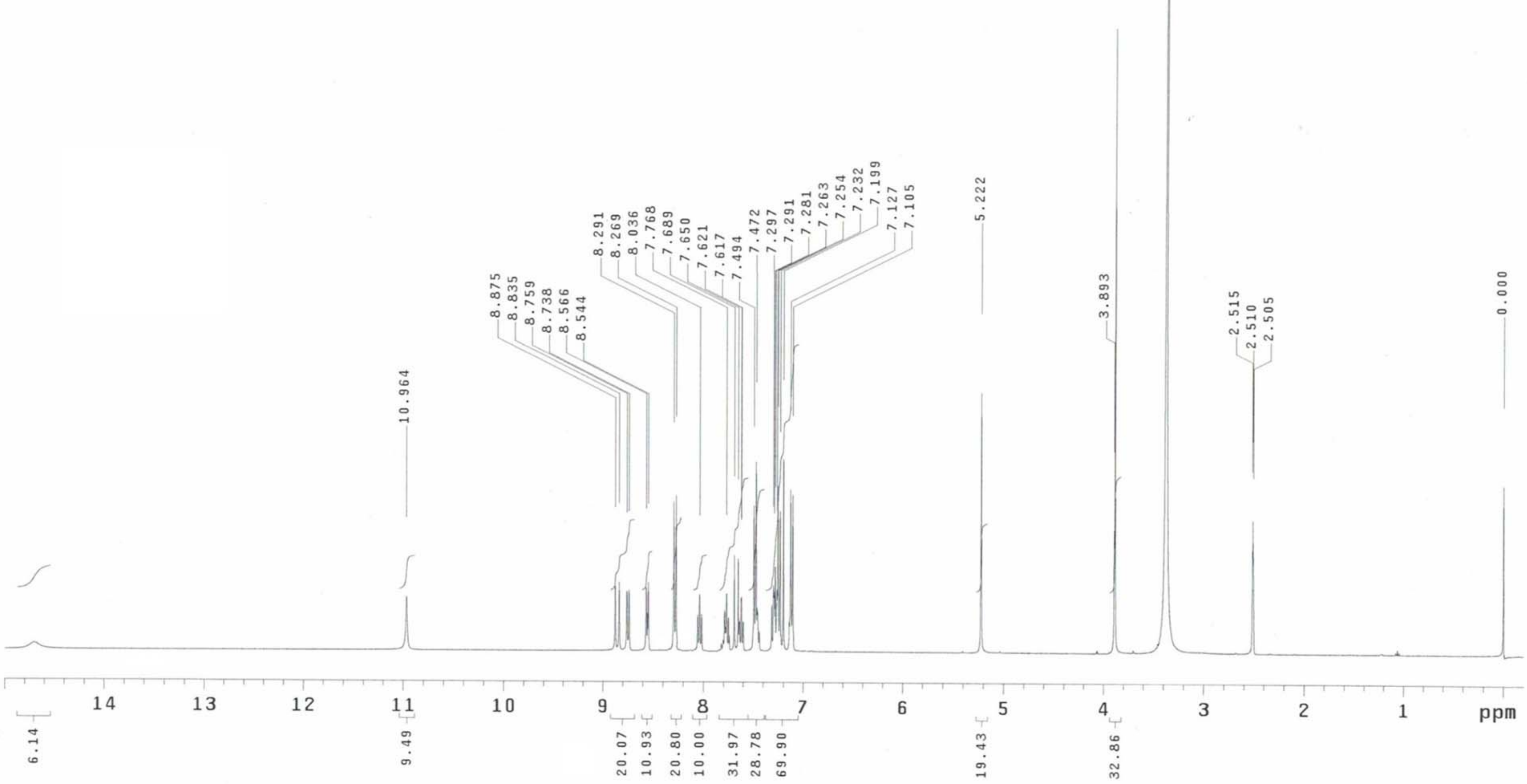

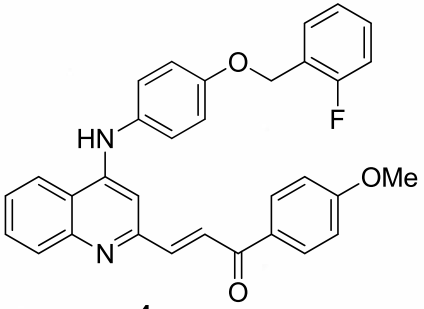
HN
O
F
OMe
N
O

4c

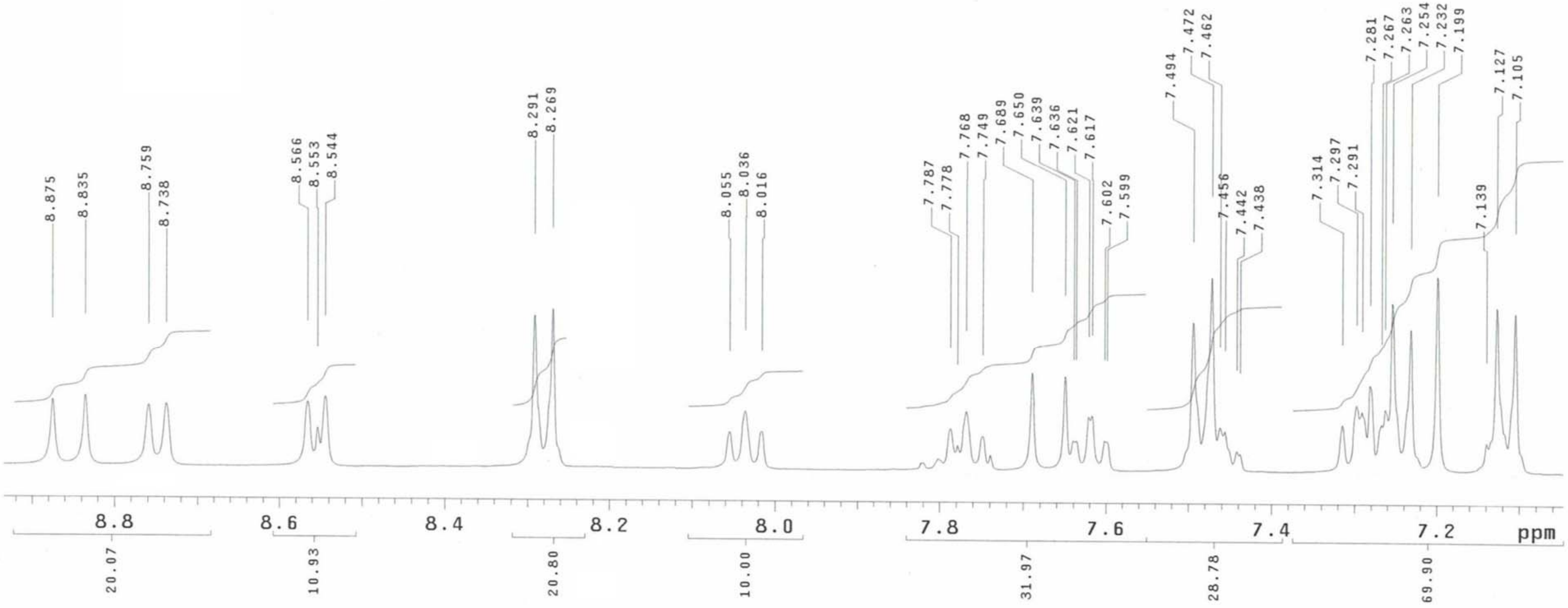
8.875
8.835
8.759
8.738
8.566
8.553
8.544
8.291
8.269
8.055
8.036
8.016
7.787
7.778
7.768
7.749
7.689
7.650
7.639
7.636
7.621
7.617
7.602
7.599
7.494
7.472
7.462
7.456
7.442
7.438
7.314
7.297
7.291
7.281
7.267
7.263
7.254
7.232
7.199
7.139
7.127
7.105
8.8
8.6
8.4
8.2
8.0
7.8
7.6
7.4
7.2
ppm
20.07
10.93
20.80
10.00
31.97
28.78
69.90

YCY-5458

Pulse Sequence: s2pul

Mercury-400BB "MercuryPlus400"
Date: Sep 3 2014
Solvent: DMSO
Ambient temperature
Total 5152 repetitions

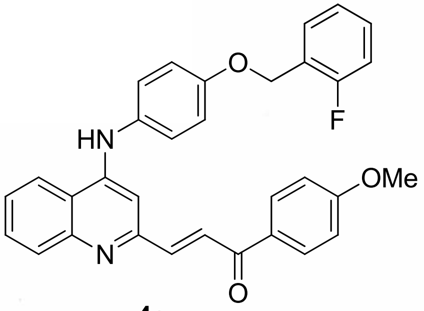

4c

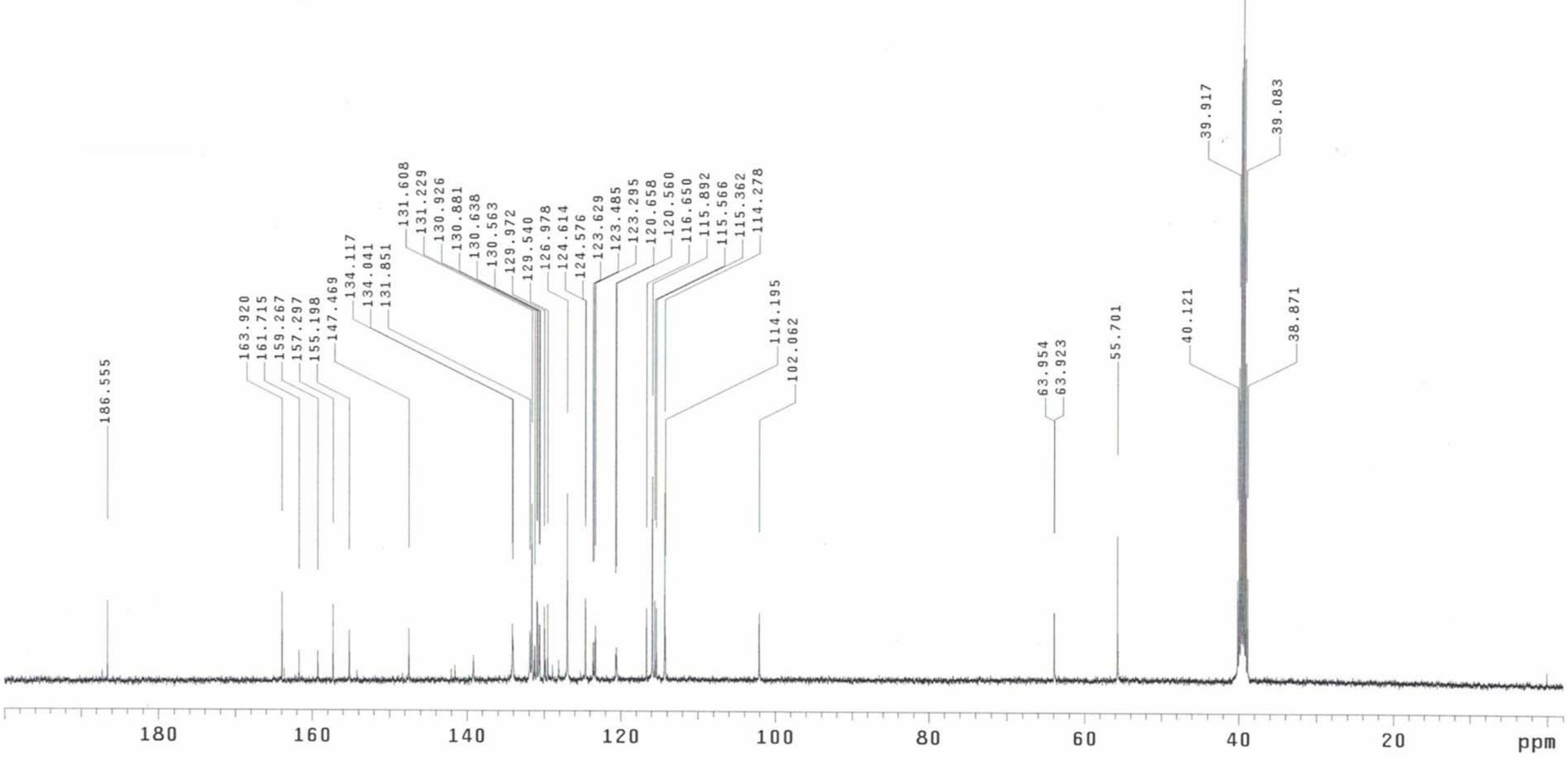

YCY-5466

Pulse Sequence: s2pul
Mercury-400BB "MercuryPlus400"
Date: Jan 22 2015
Solvent: DMSO
Ambient temperature
Total 32 repetitions

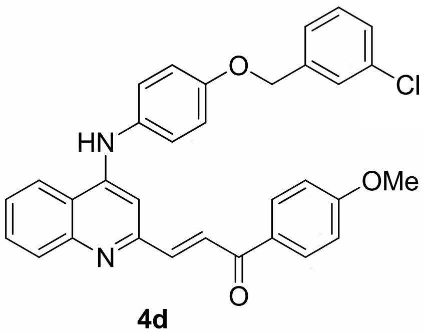

4d

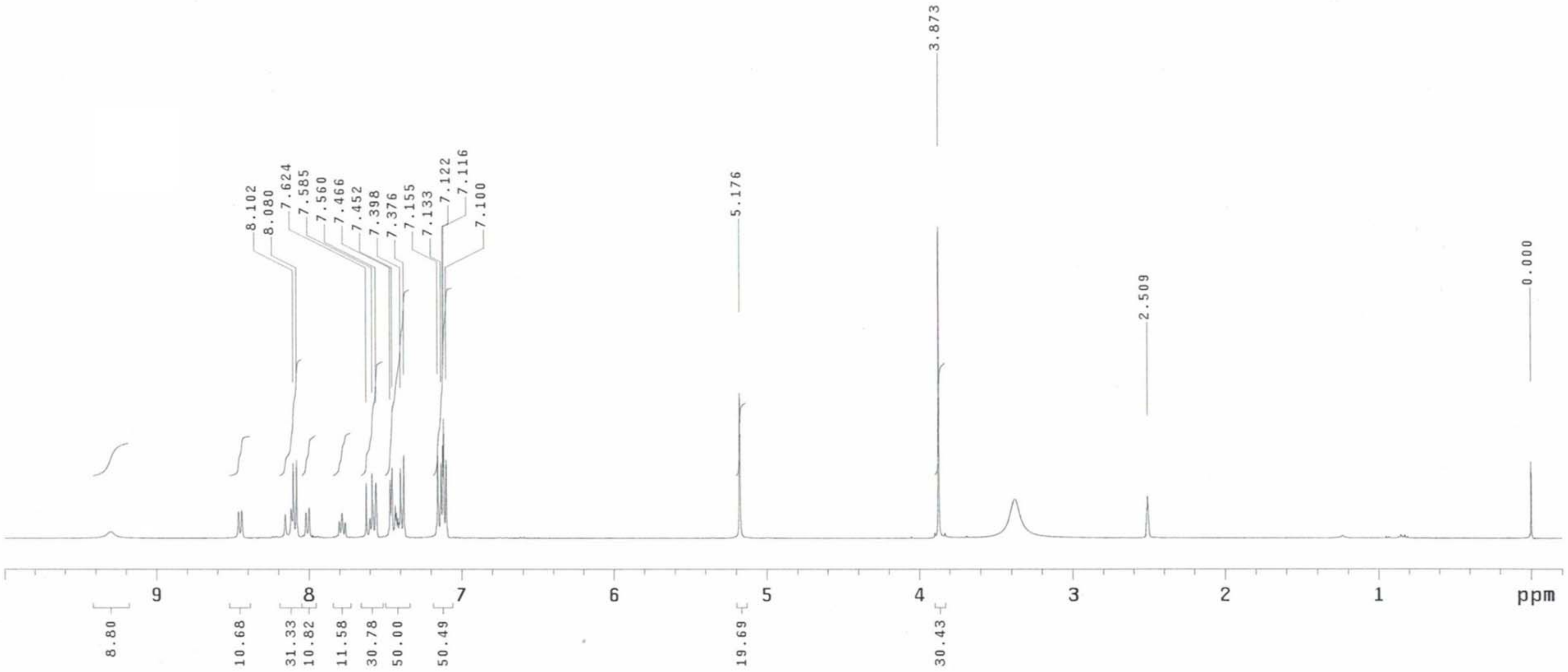

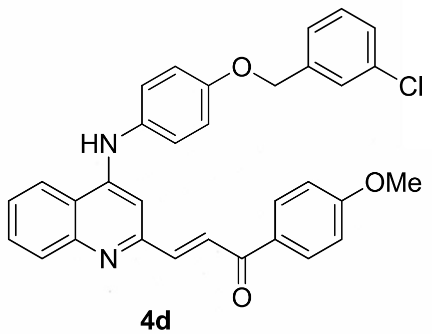
O
Cl
HN
OMe
N
O

4d

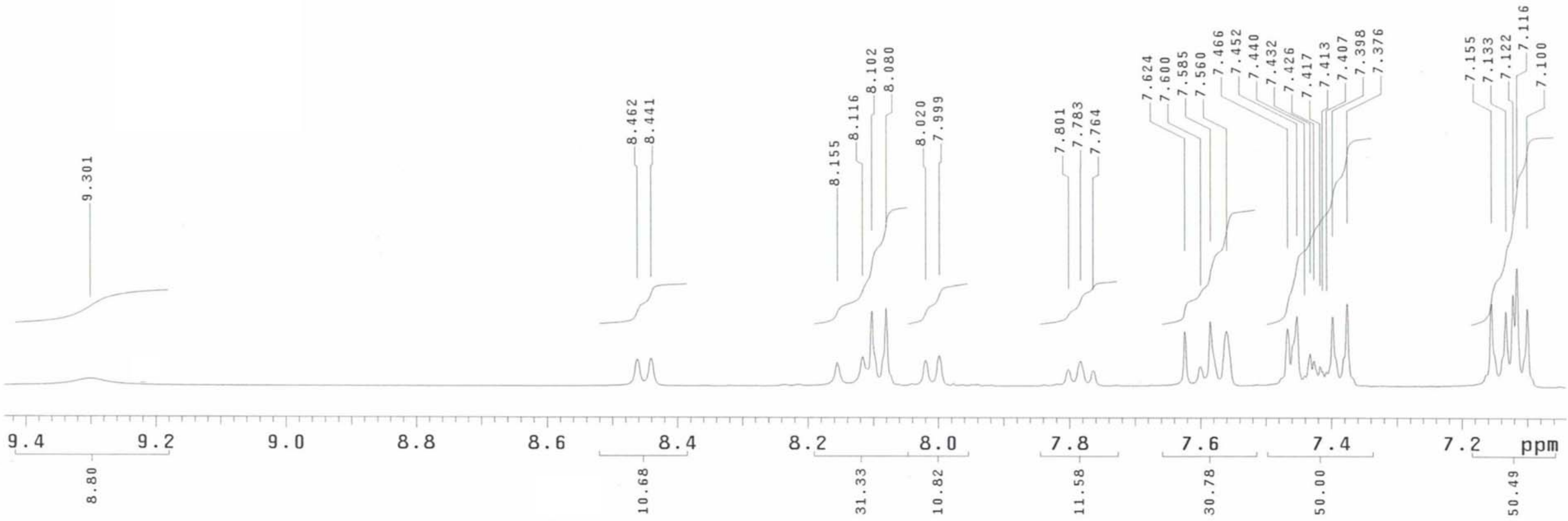
9.301
8.462
8.441
8.155
8.116
8.102
8.080
8.020
7.999
7.801
7.783
7.764
7.624
7.600
7.585
7.560
7.466
7.452
7.440
7.432
7.426
7.417
7.413
7.407
7.398
7.376
7.155
7.133
7.122
7.116
7.100
9.4
9.2
9.0
8.8
8.6
8.4
8.2
8.0
7.8
7.6
7.4
7.2
ppm
8.80
10.68
31.33
10.82
11.58
30.78
50.00
50.49

YCY-5466

Pulse Sequence: s2pul

Mercury-400BB "MercuryPlus400"
Date: Jan 22 2015
Solvent: DMSO
Ambient temperature
Total 5760 repetitions

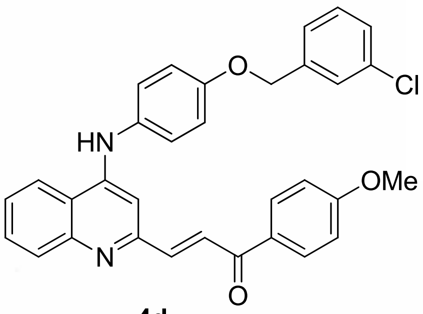

4d

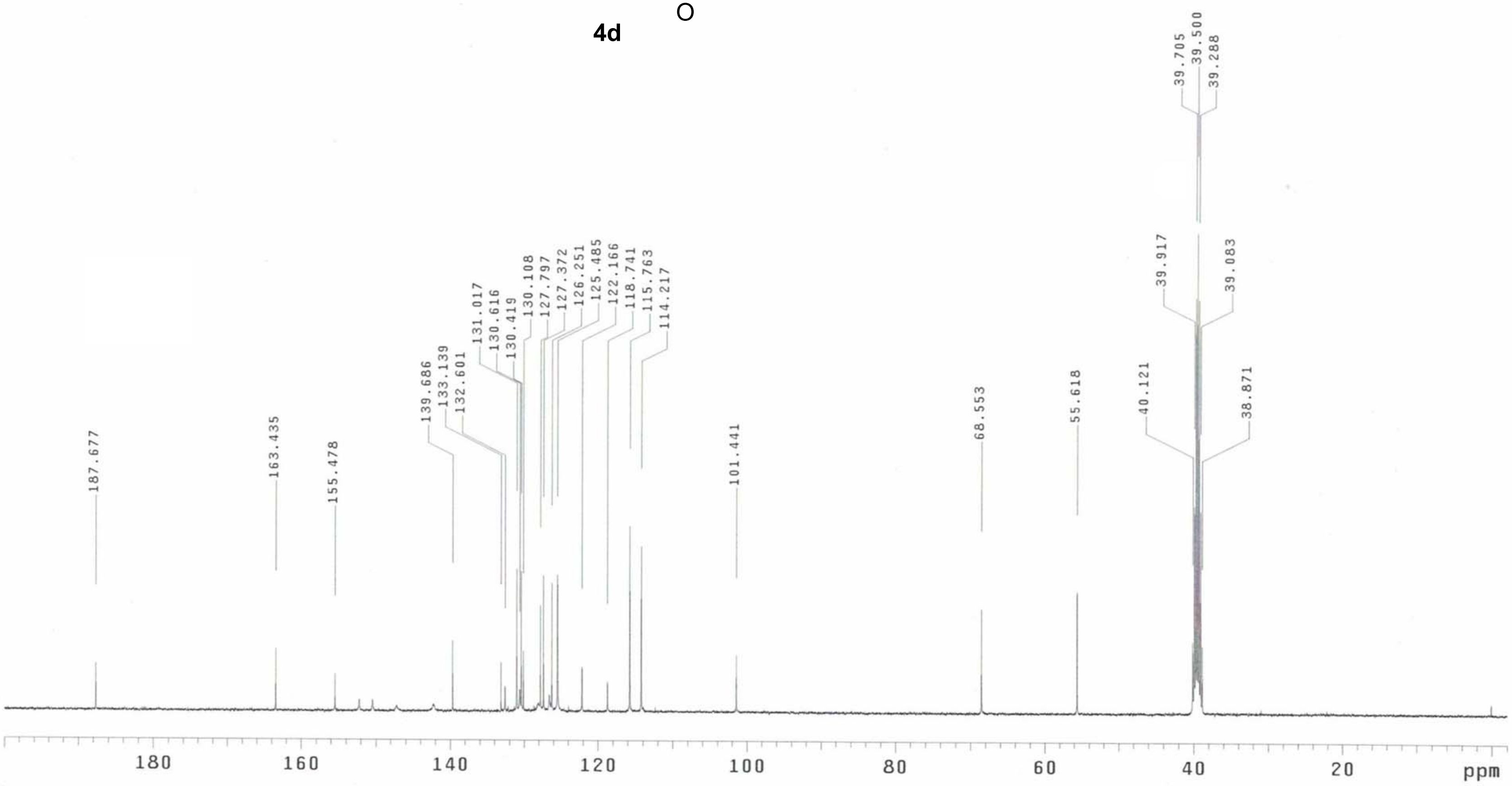

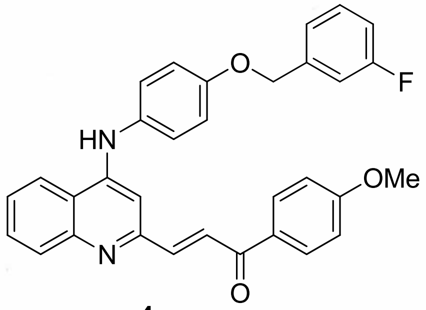
HN
OMe
O
F
N

4e

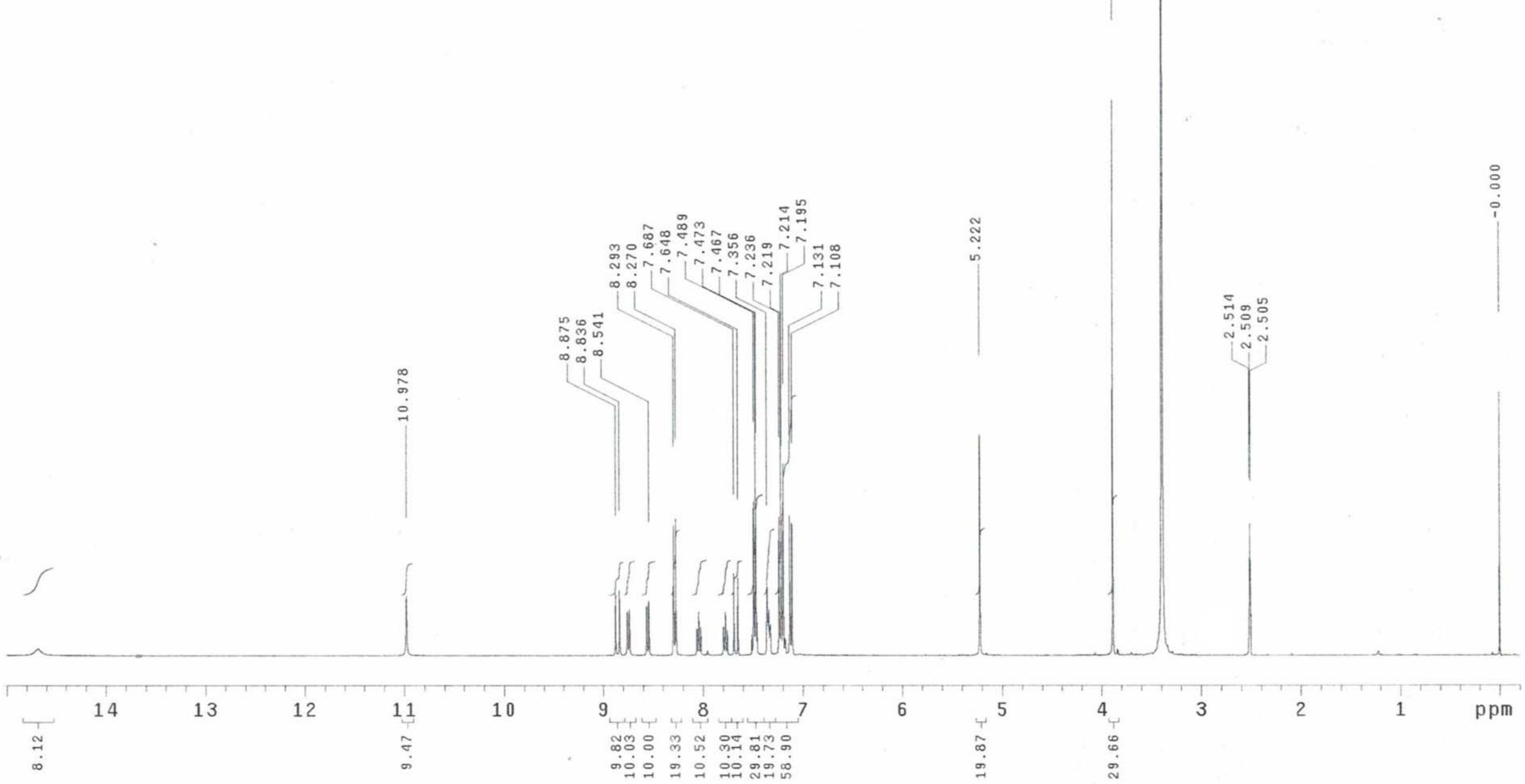
YCY-5454
Pulse Sequence: s2pul
UNITYplus-400 "unity400"
Date: Aug 25 2014
Solvent: DMSO
Ambient temperature
Total 64 repetitions
10.978
8.875
8.836
8.541
8.293
8.270
7.687
7.648
7.489
7.473
7.467
7.356
7.236
7.219
7.214
7.195
7.131
7.108
5.222
3.886
3.387
2.514
2.509
2.505
-0.000
14
13
12
11
10
9
8
7
6
5
4
3
2
1
ppm
8.12
9.47
9.82
10.03
10.00
19.33
10.52
10.30
10.14
29.81
19.73
58.90
19.87
29.66

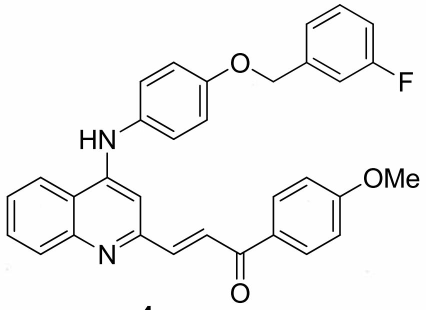

4e

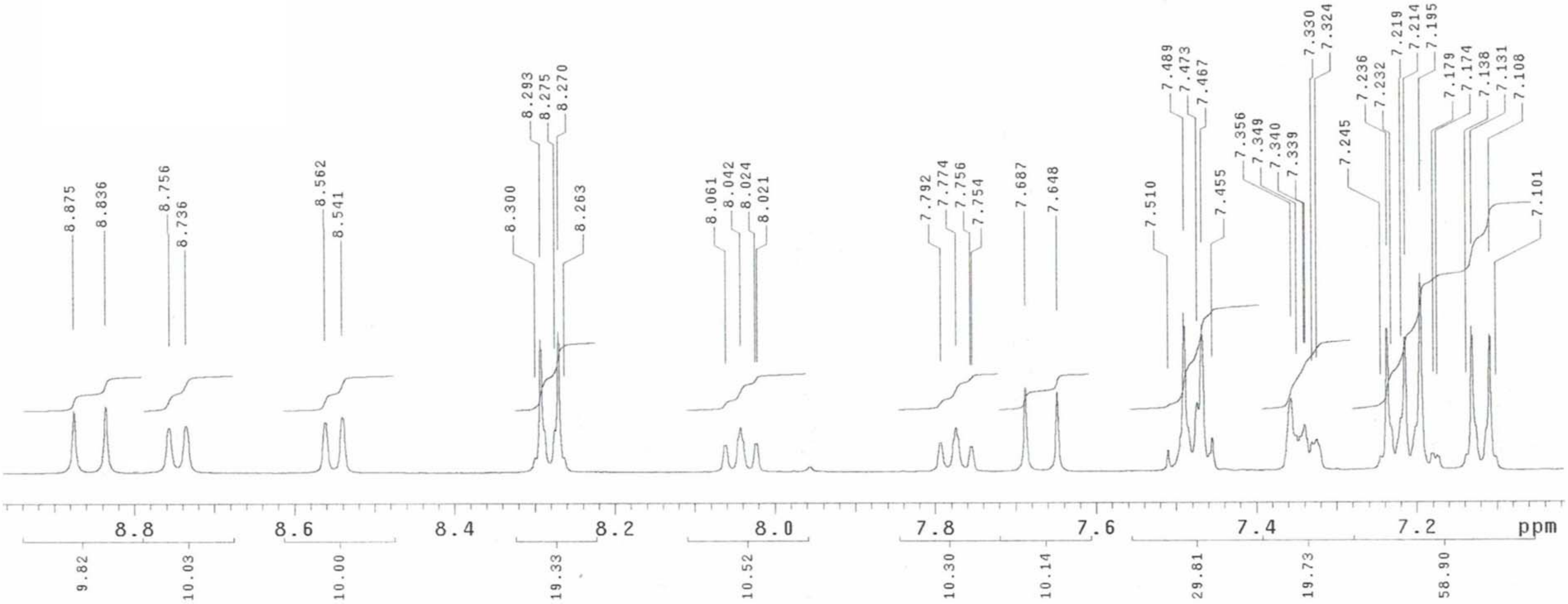

YCY-5454

Pulse Sequence: s2pul
UNITYplus-400 "unity400"
Date: Aug 25 2014
Solvent: DMSO
Ambient temperature
Total 13568 repetitions

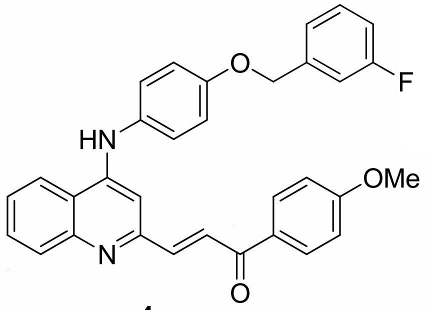

4e

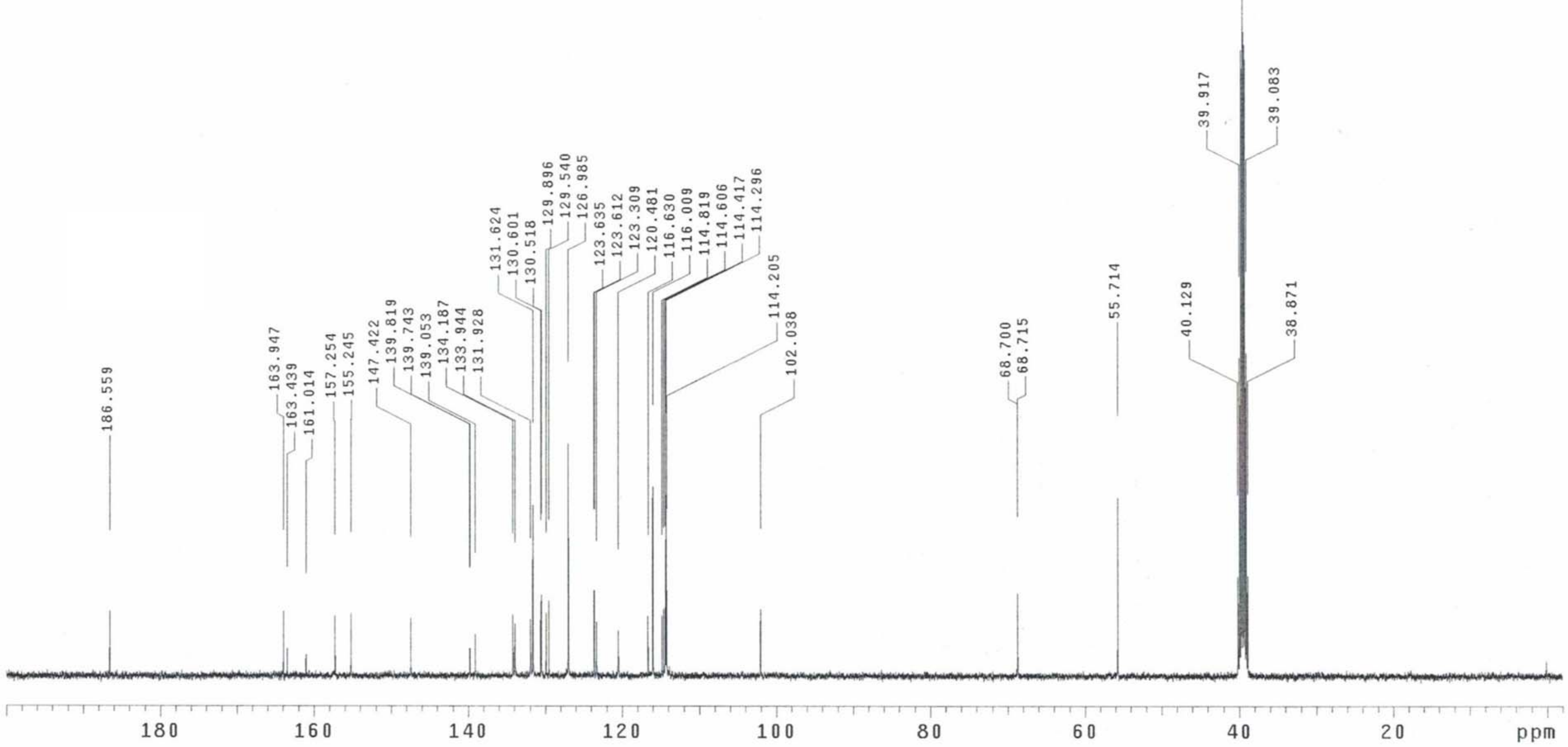

YCY-5453

Pulse Sequence: s2pul

UNITYplus-400 "unity400"
Date: Aug 22 2014
Solvent: DMSO
Ambient temperature
Total 64 repetitions

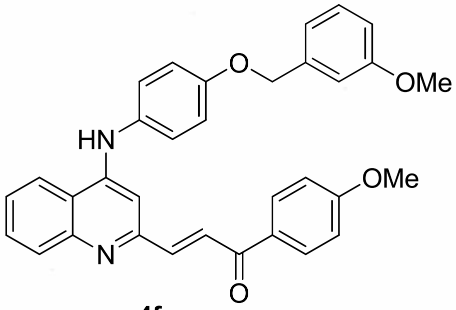

4f

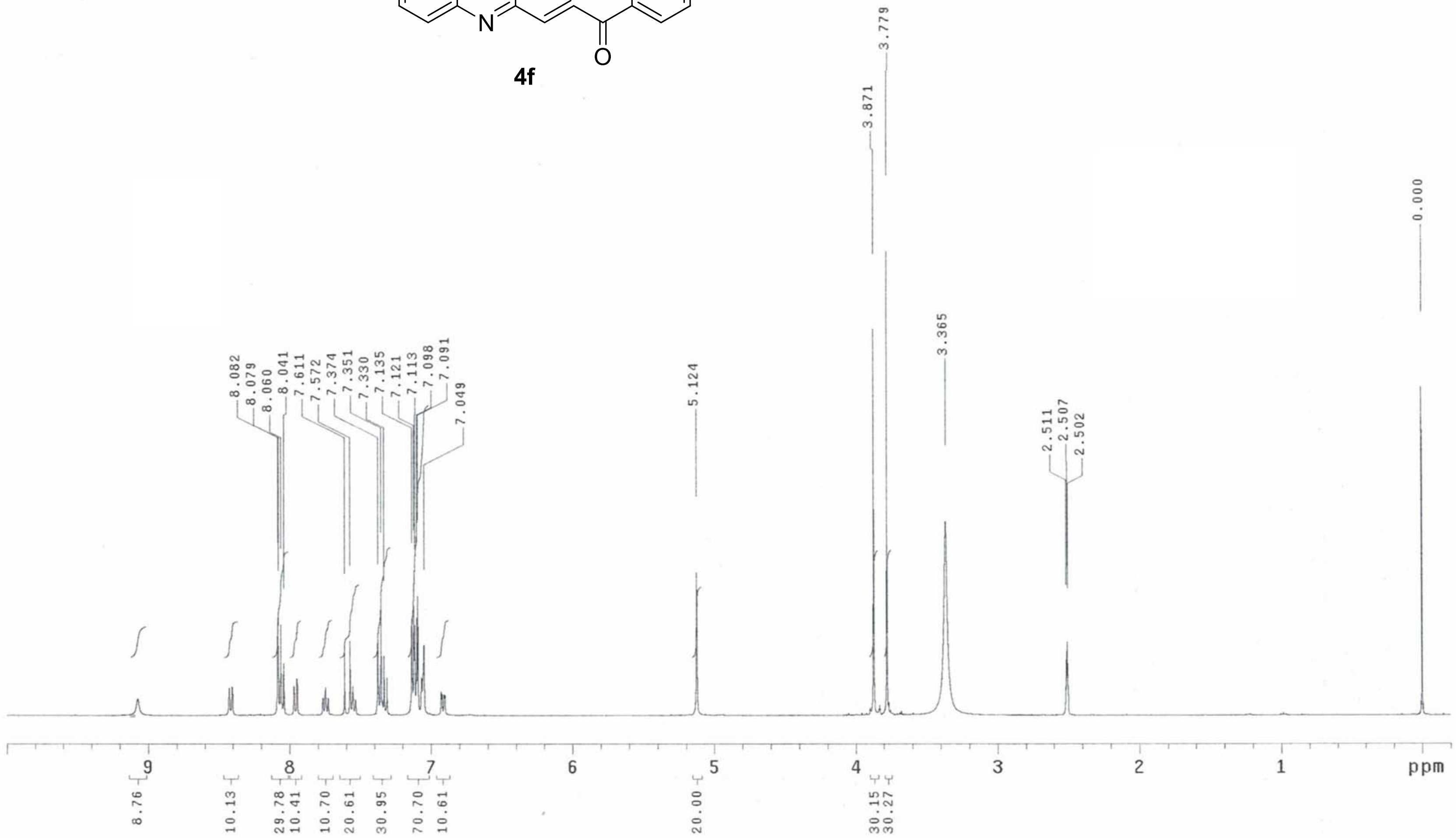

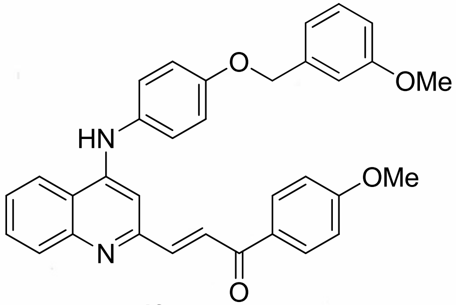
HN
N
O
OMe
OMe
O

4f

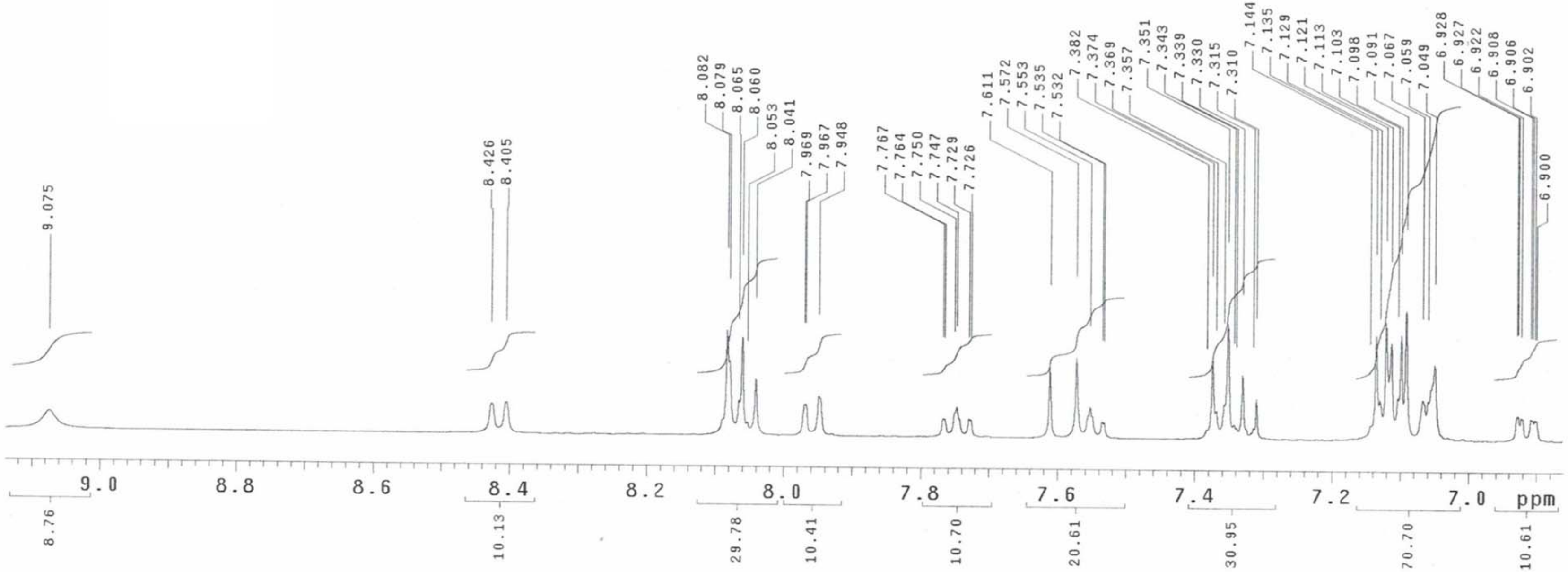
9.075
8.426
8.405
8.082
8.079
8.065
8.060
8.053
8.041
7.969
7.967
7.948
7.767
7.764
7.750
7.747
7.729
7.726
7.611
7.572
7.553
7.535
7.532
7.382
7.374
7.369
7.357
7.351
7.343
7.339
7.330
7.315
7.310
7.144
7.135
7.129
7.121
7.113
7.103
7.098
7.091
7.067
7.059
7.049
6.928
6.927
6.922
6.908
6.906
6.902
6.900
9.0
8.8
8.6
8.4
8.2
8.0
7.8
7.6
7.4
7.2
7.0
ppm
8.76
10.13
29.78
10.41
10.70
20.61
30.95
70.70
10.61

YCY-5453

Pulse Sequence: s2pul

UNITYplus-400 "unity400"
Date: Aug 22 2014
Solvent: DMSO
Ambient temperature
Total 64000 repetitions

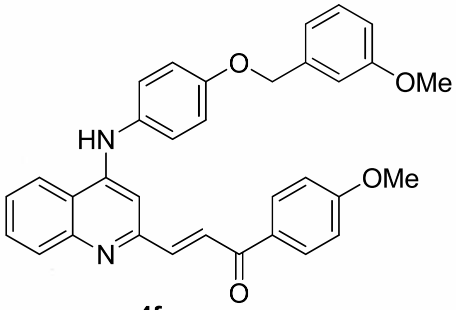

4f

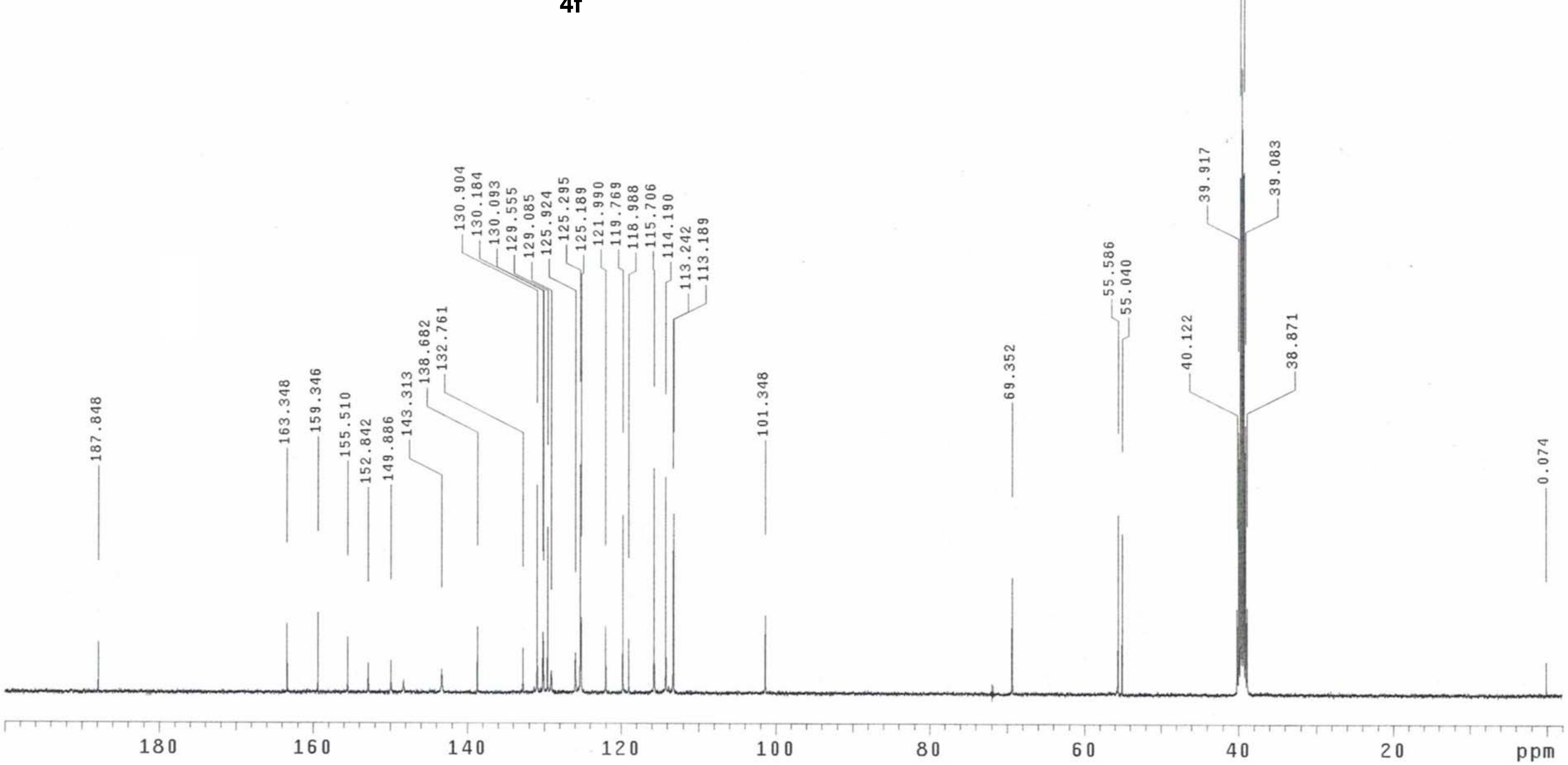

YCY-5460

Pulse Sequence: s2pul
Mercury-400BB "MercuryPlus400"
Date: Sep 3 2014
Solvent: DMSO
Ambient temperature
Total 48 repetitions

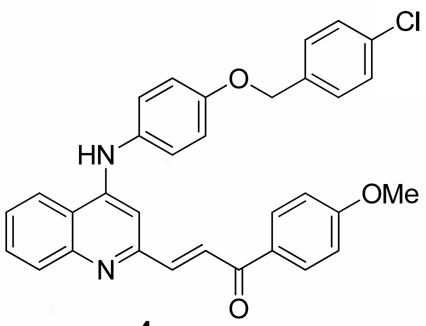

4g

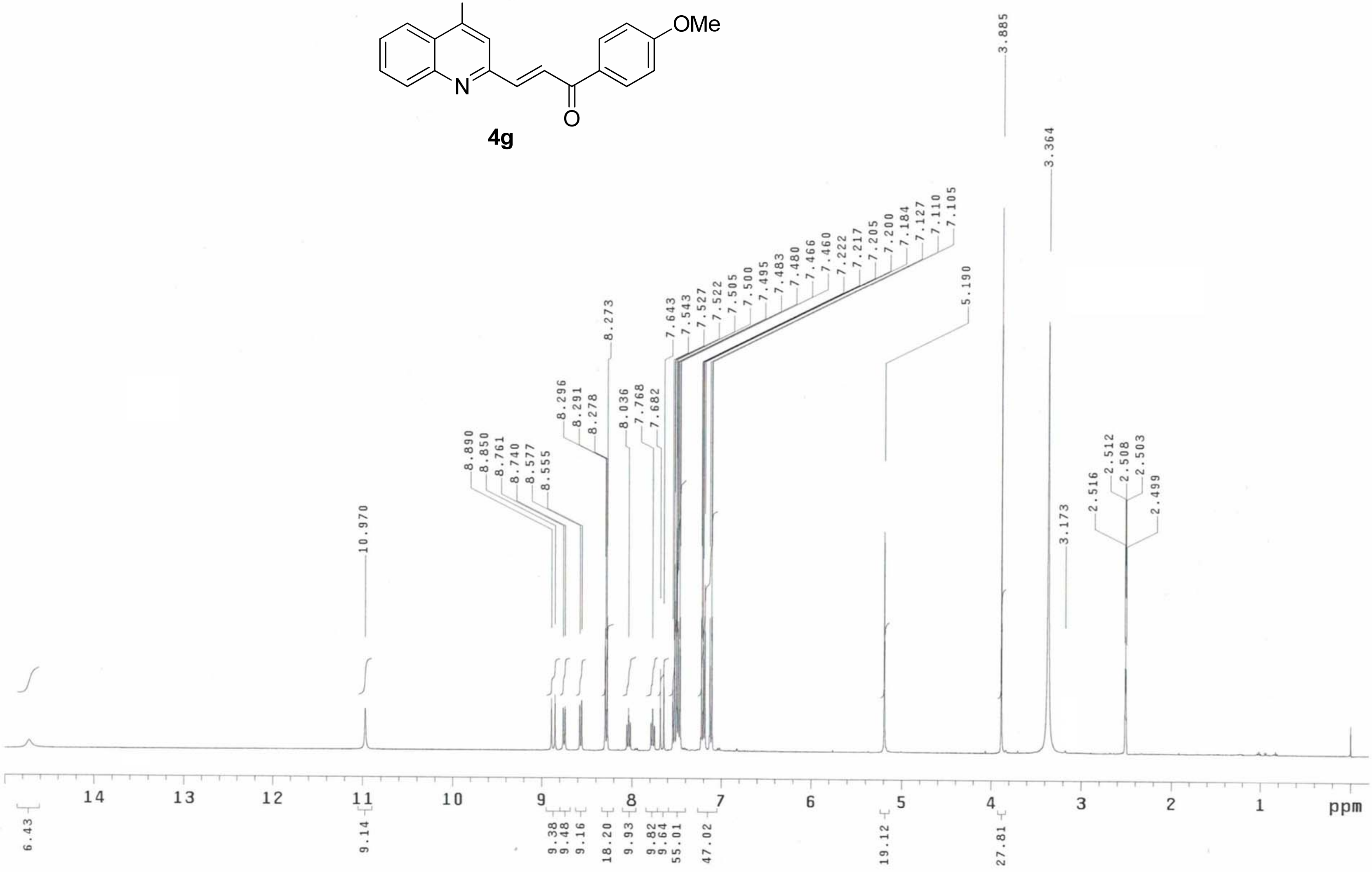

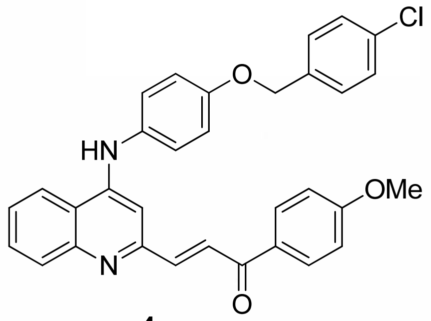

4g

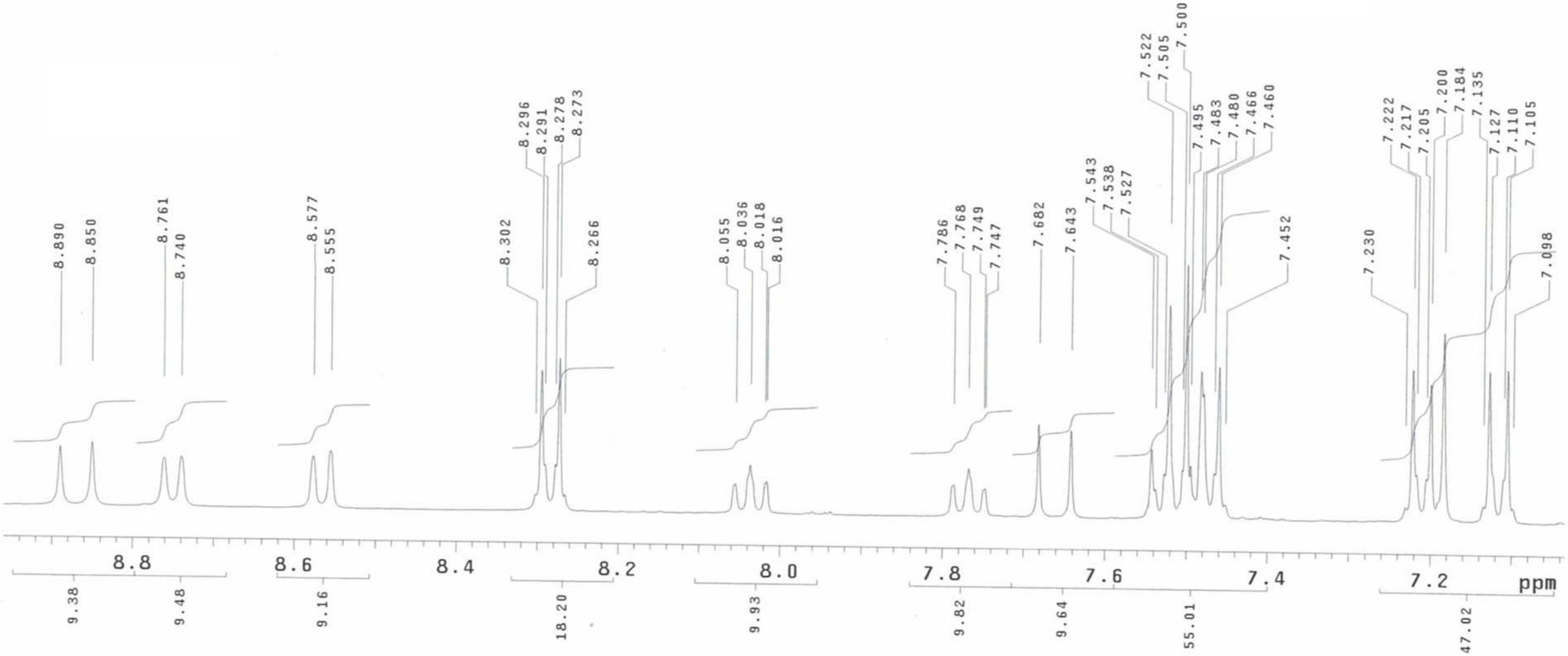

YCY-5460

Pulse Sequence: s2pul
Mercury-400BB "MercuryPlus400"
Date: Sep 3 2014
Solvent: DMSO
Ambient temperature
Total 4752 repetitions

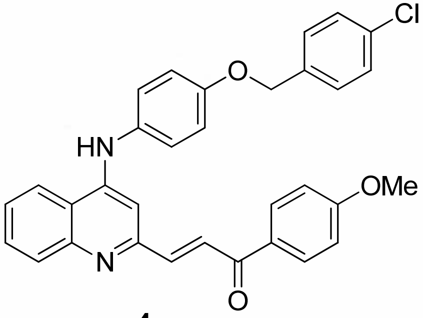

4g

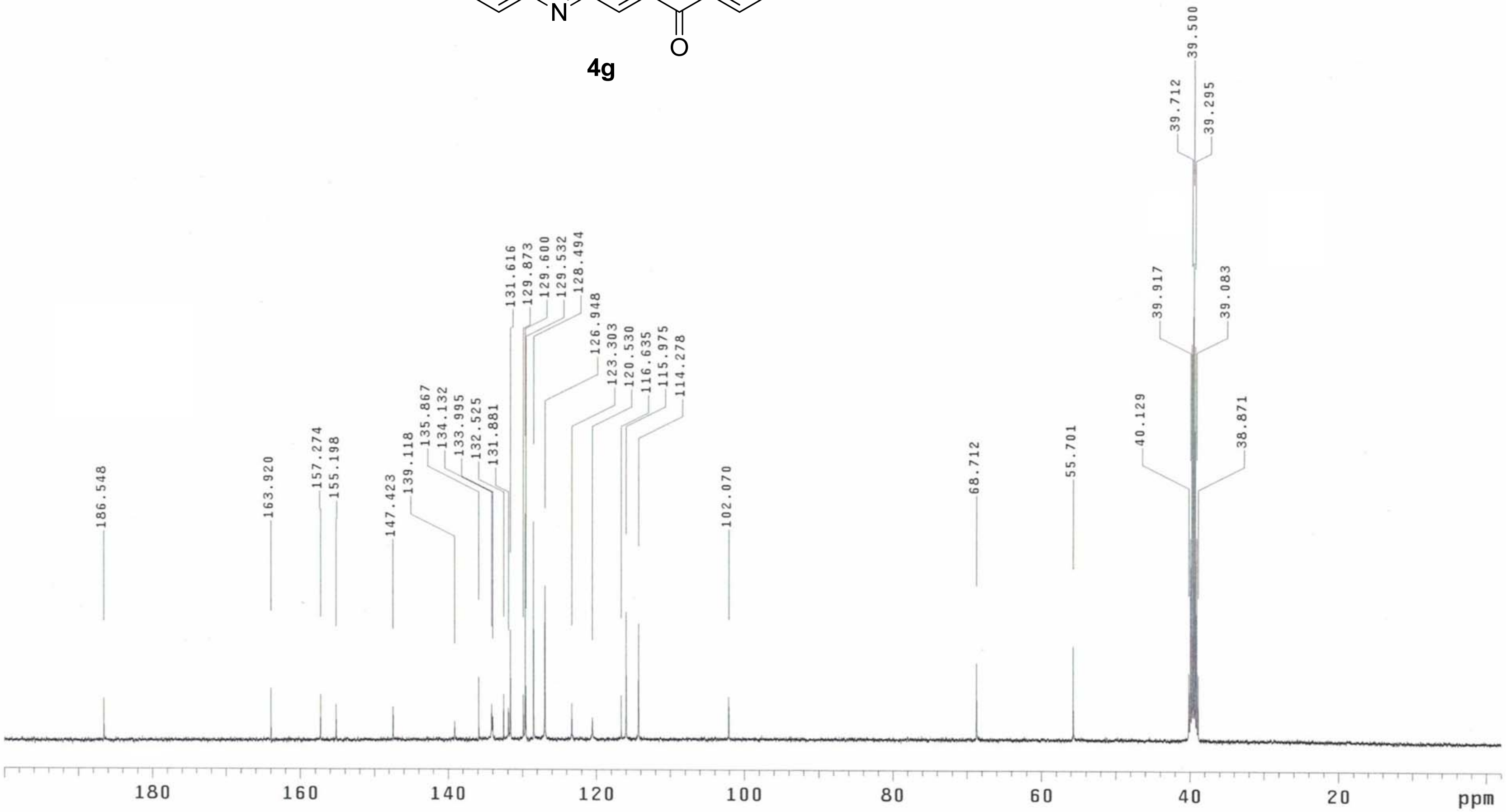

YCY-5456

Pulse Sequence: s2pul

UNITYplus-400 "unity400"
Date: Aug 25 2014
Solvent: DMSO
Ambient temperature
Total 64 repetitions

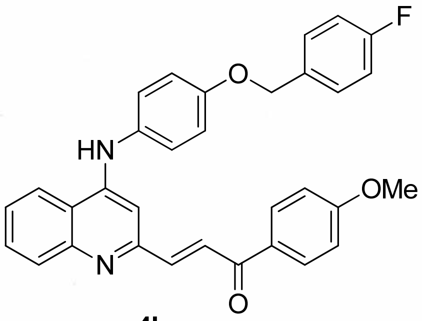

4h

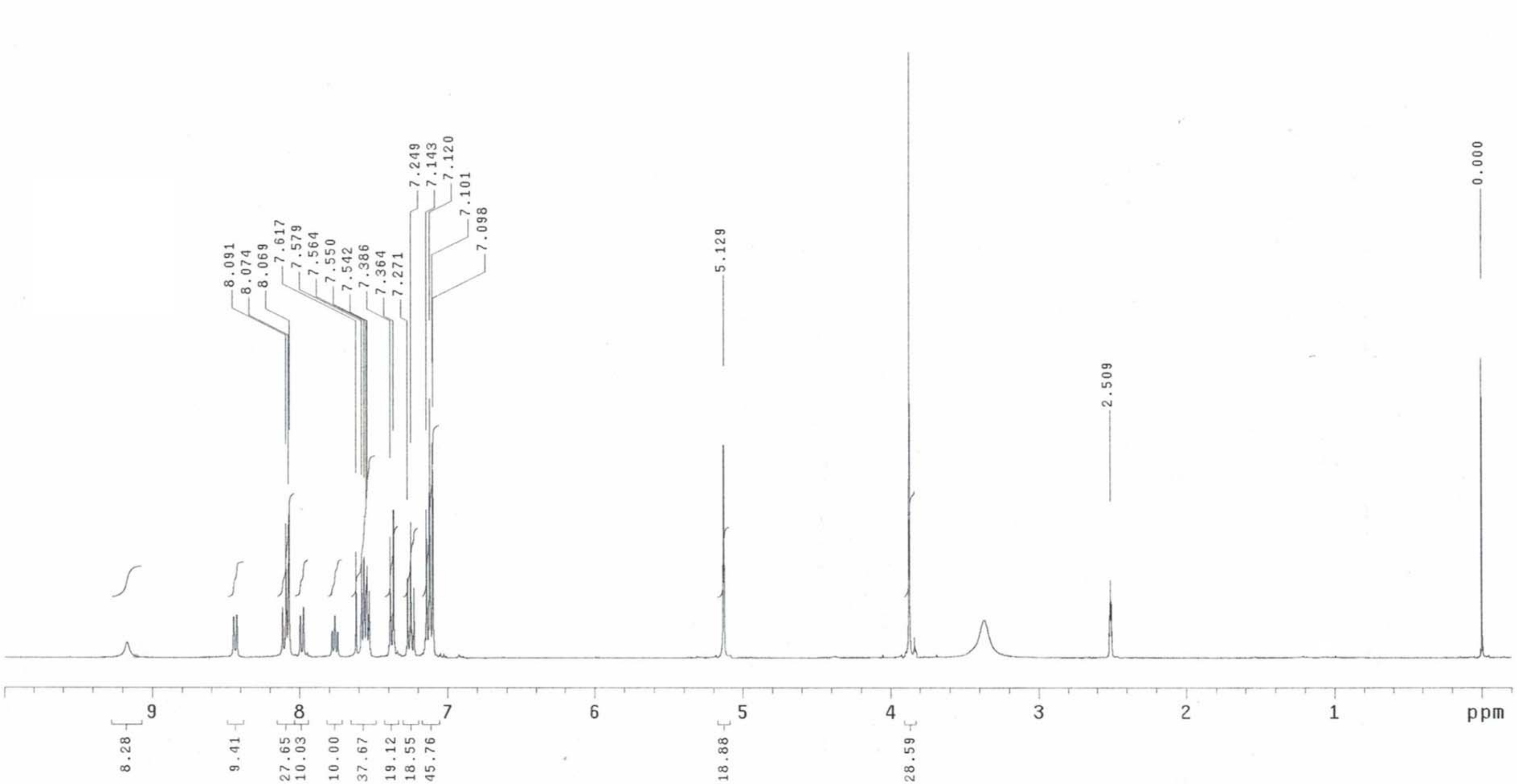

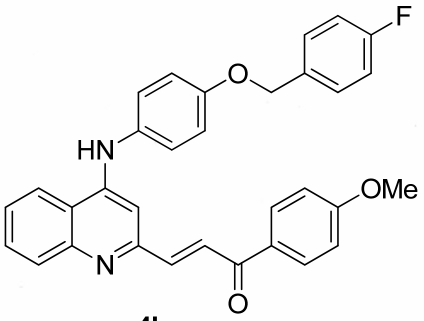
F
O
HN
OMe
N
O

4h

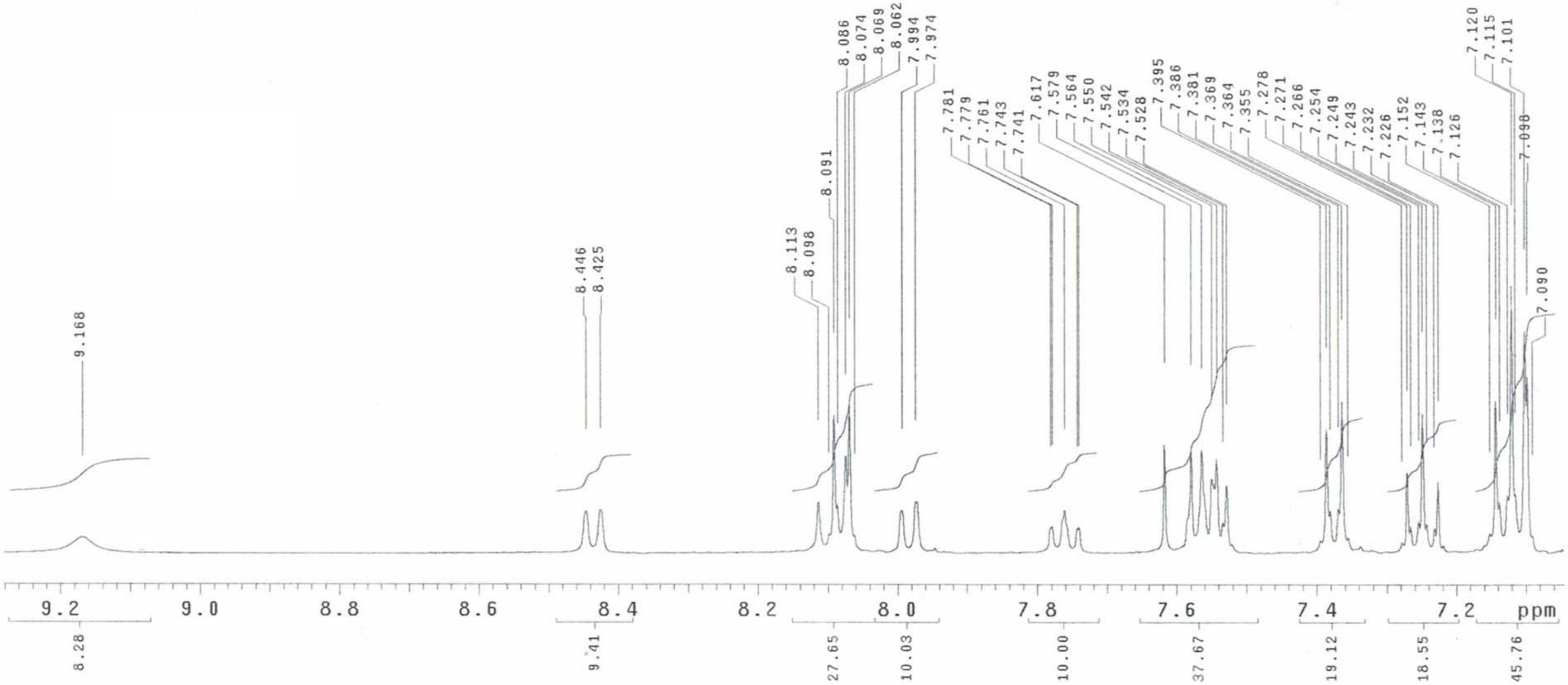
9.168
8.446
8.425
8.113
8.098
8.091
8.086
8.074
8.069
8.062
7.994
7.974
7.781
7.779
7.761
7.743
7.741
7.617
7.579
7.564
7.550
7.542
7.534
7.528
7.395
7.386
7.381
7.369
7.364
7.355
7.278
7.271
7.266
7.254
7.249
7.243
7.232
7.226
7.152
7.143
7.138
7.126
7.120
7.115
7.101
7.098
7.090
9.2
9.0
8.8
8.6
8.4
8.2
8.0
7.8
7.6
7.4
7.2
ppm
8.28
9.41
27.65
10.03
10.00
37.67
19.12
18.55
45.76

YCY-5456

Pulse Sequence: s2pul

UNITYplus-400 "unity400"
Date: Aug 25 2014
Solvent: DMSO
Ambient temperature
Total 64000 repetitions

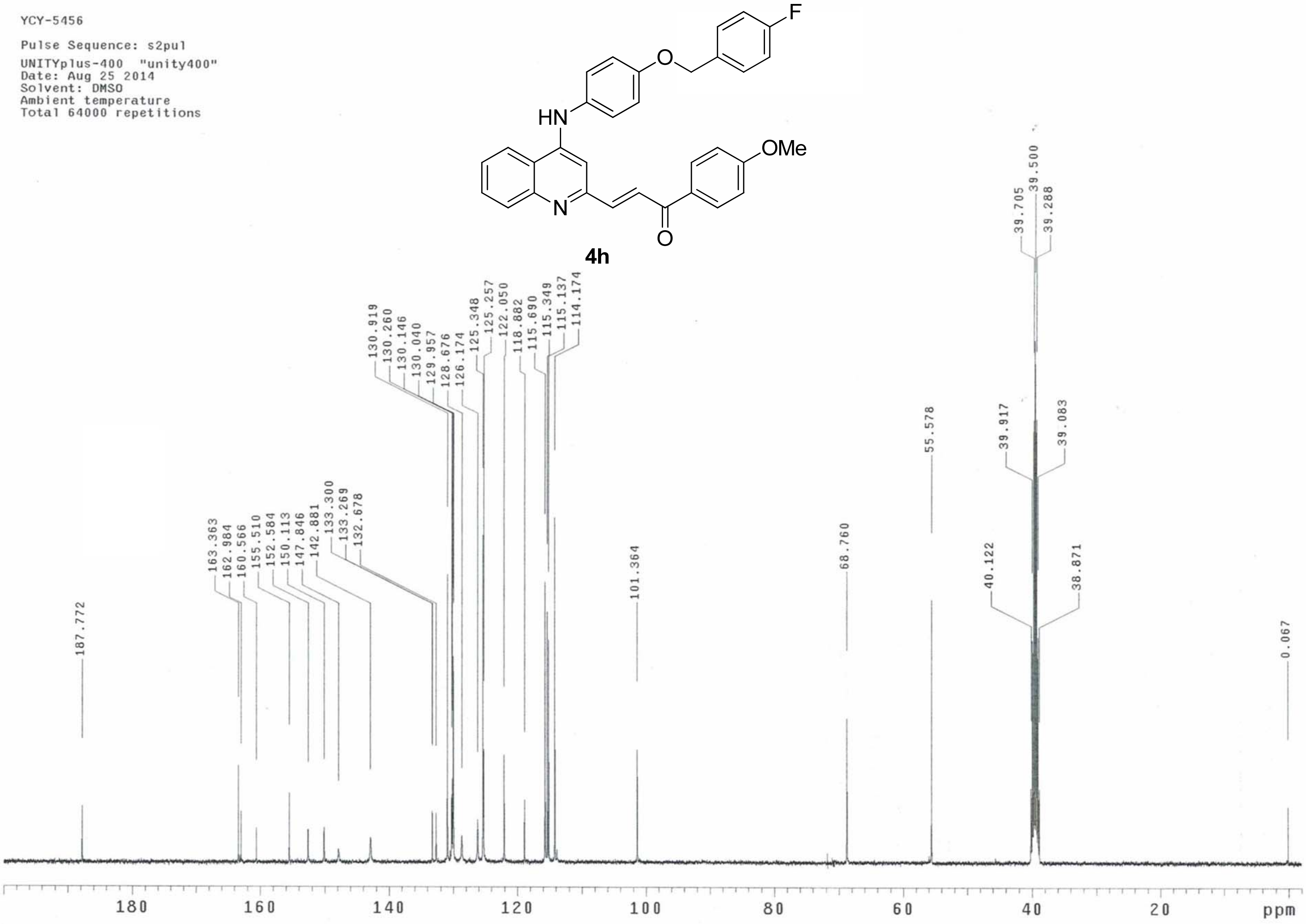

YCY-5839

Pulse Sequence: s2pul
Mercury-400BB "MerPlus400"
Date: Oct 5 2018
Solvent: dmso
Ambient temperature
Total 32 repetitions

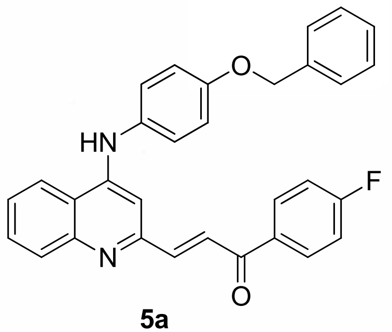

5a

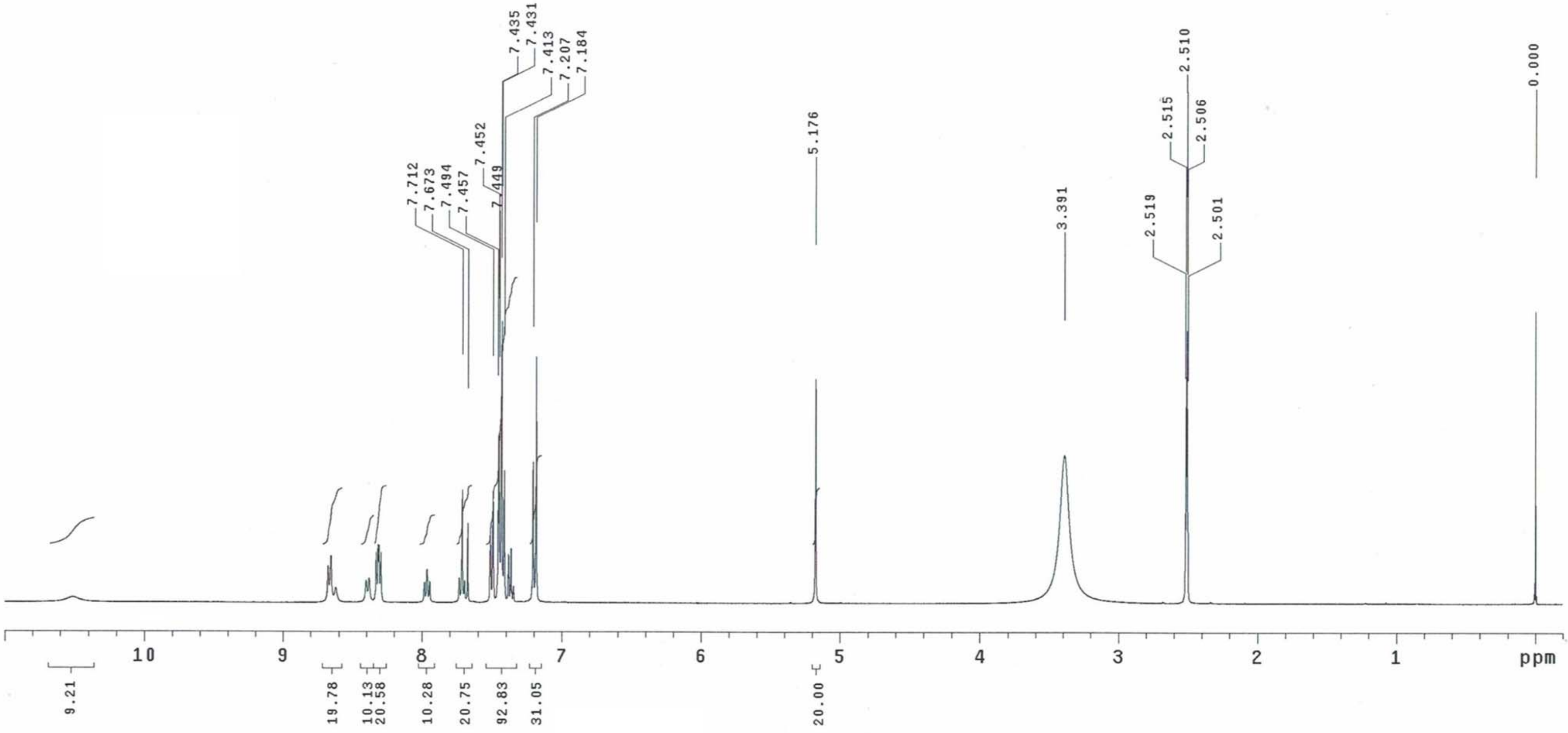

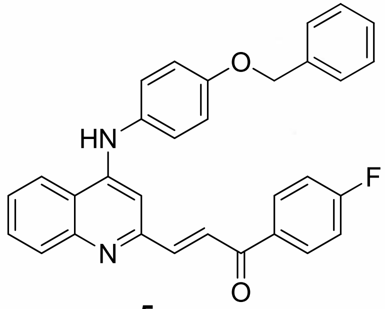
HN
O
F
N
O

5a

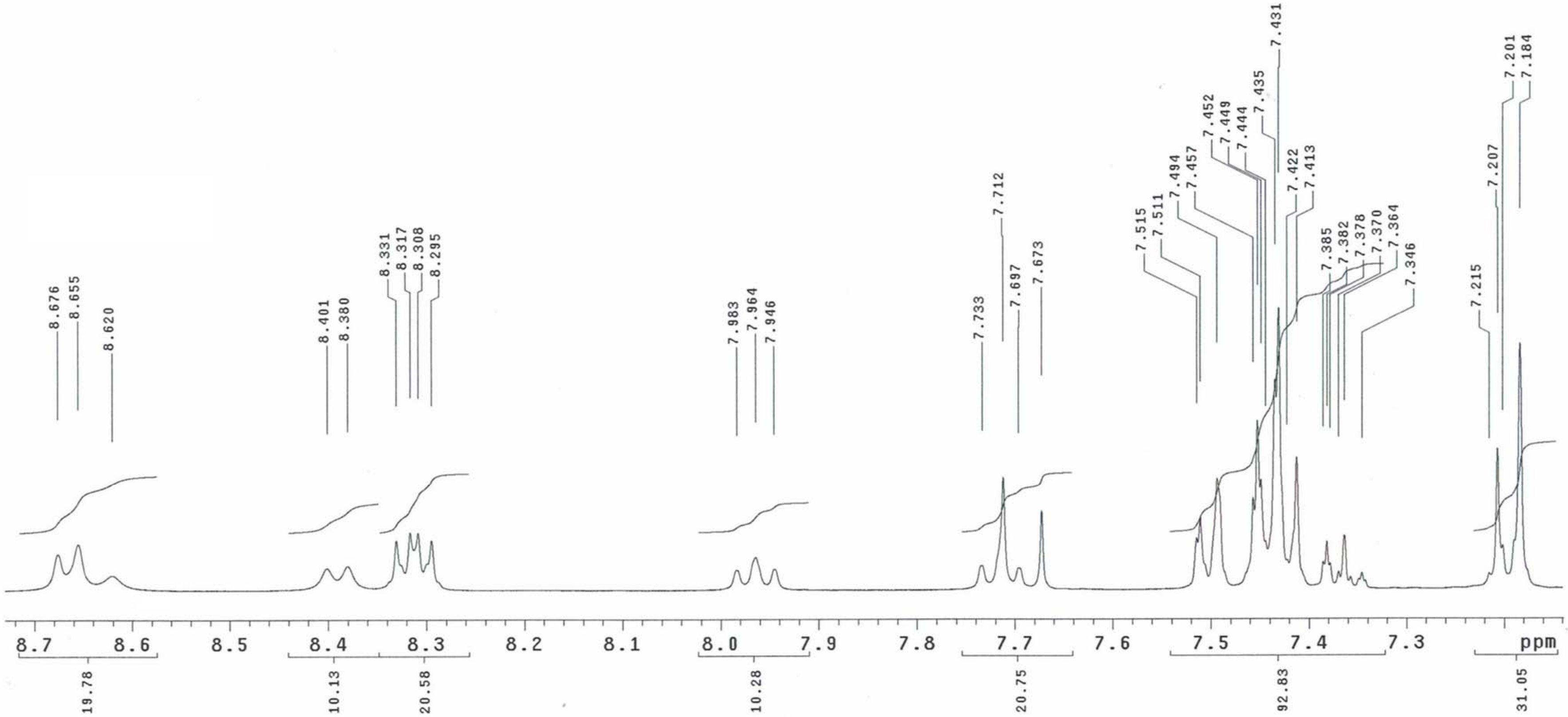
8.676
8.655
8.620
8.401
8.380
8.331
8.317
8.308
8.295
7.983
7.964
7.946
7.733
7.712
7.697
7.673
7.515
7.511
7.494
7.457
7.452
7.449
7.444
7.435
7.431
7.422
7.413
7.385
7.382
7.378
7.370
7.364
7.346
7.215
7.207
7.201
7.184
8.7
8.6
8.5
8.4
8.3
8.2
8.1
8.0
7.9
7.8
7.7
7.6
7.5
7.4
7.3
ppm
19.78
10.13
20.58
10.28
20.75
92.83
31.05

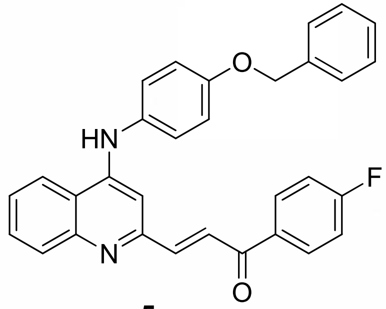

5a

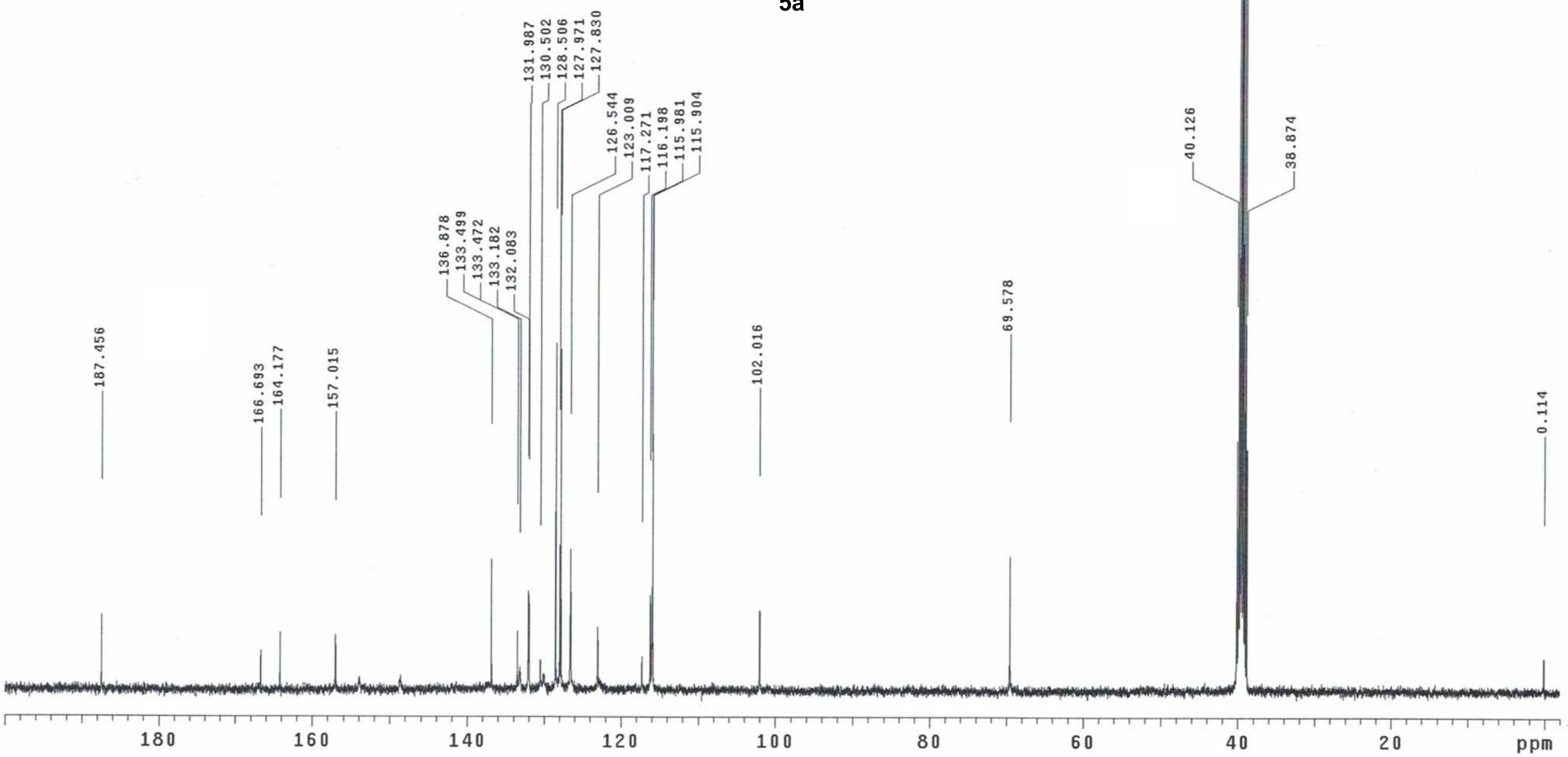

YCY-5839
Pulse Sequence: s2pul
Mercury-400BB "MerPlus400"
Date: Oct 5 2018
Solvent: dmso
Ambient temperature
Total 4864 repetitions
187.456
166.693
164.177
157.015
136.878
133.499
133.472
133.182
132.083
131.987
130.502
128.506
127.971
127.830
126.544
123.009
117.271
116.198
115.981
115.904
102.016
69.578
40.126
39.916
39.710
39.500
39.290
39.084
38.874
0.114
180
160
140
120
100
80
60
40
20
ppm

YCY-5832

Pulse Sequence: s2pul
UNITYplus-400 "unity400"
Date: Jul 2 2018
Solvent: DMSO
Ambient temperature
Total 32 repetitions

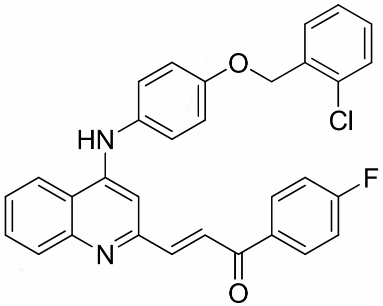

5b

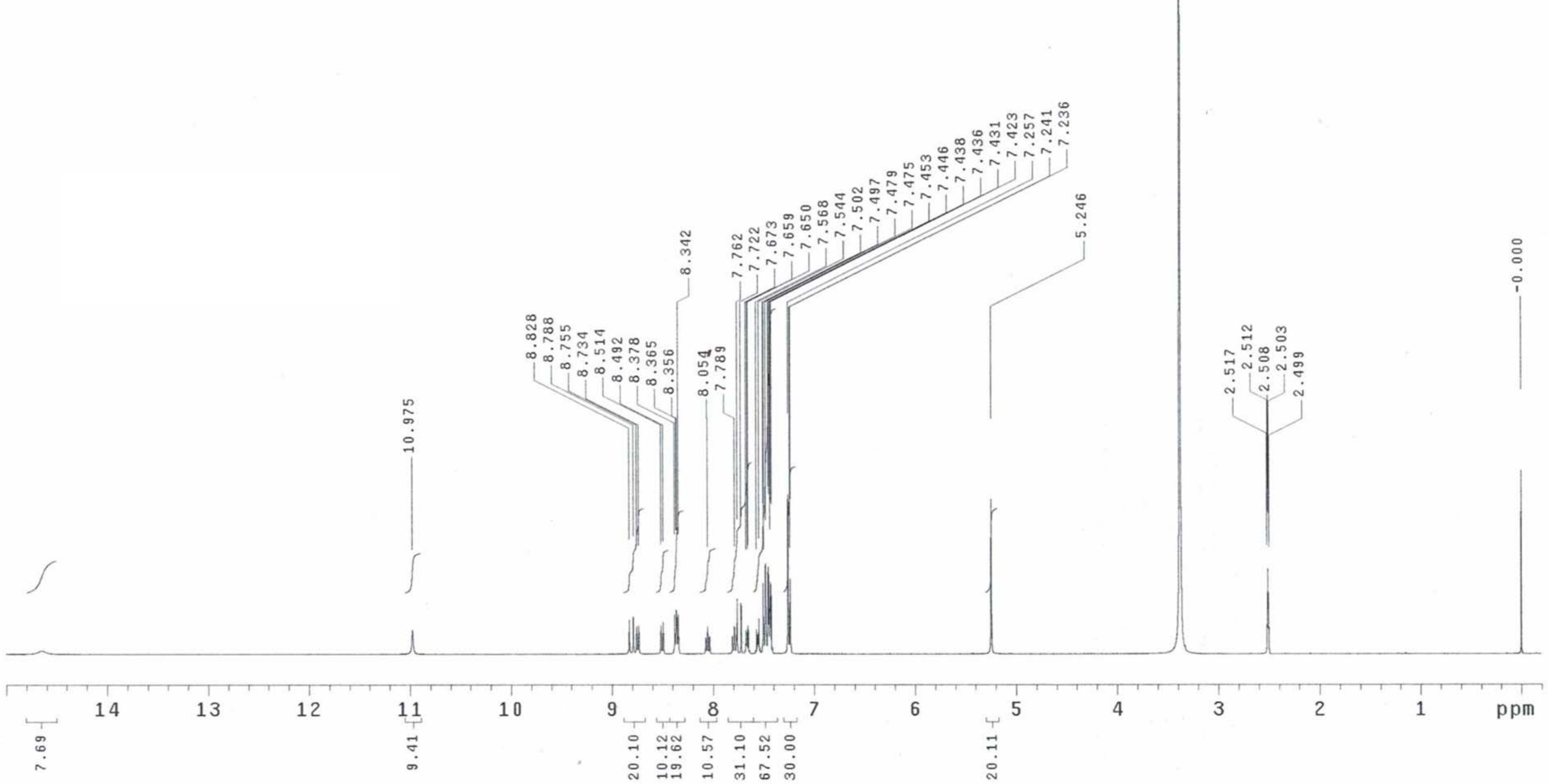

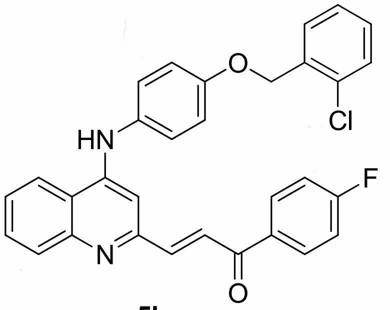
HN
O
Cl
F
N
O

5b

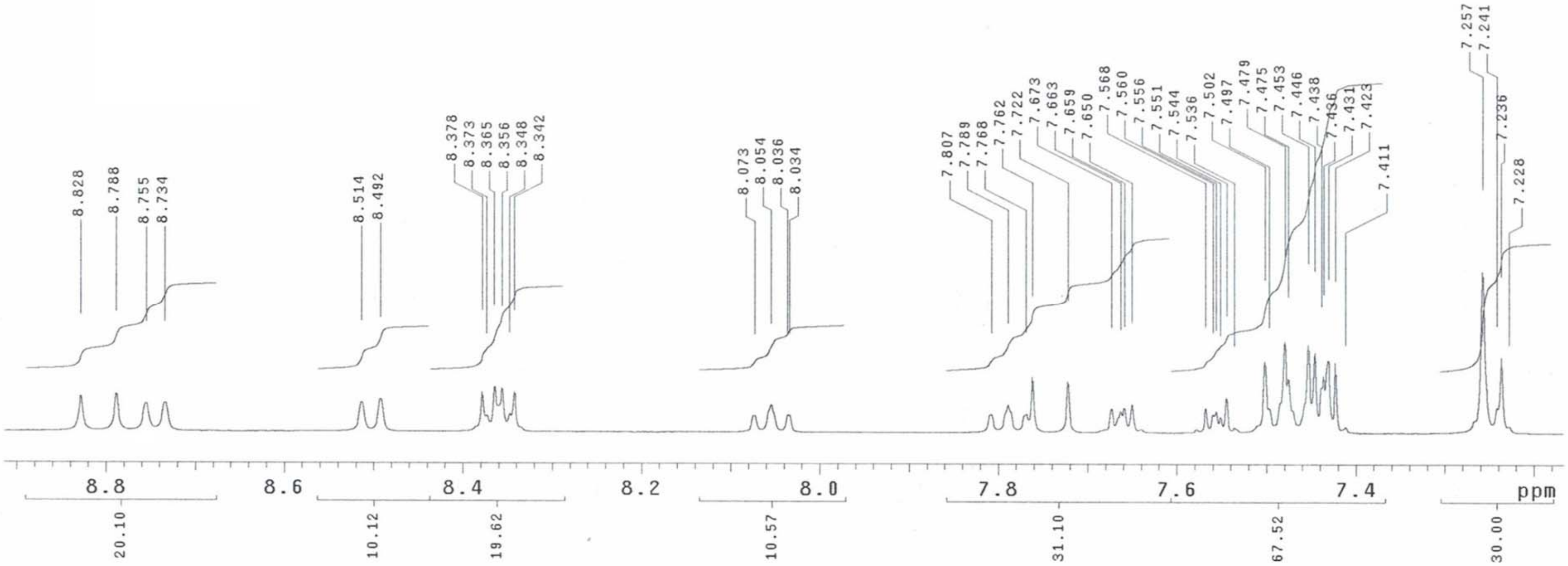
8.828
8.788
8.755
8.734
8.514
8.492
8.378
8.373
8.365
8.356
8.348
8.342
8.073
8.054
8.036
8.034
7.807
7.789
7.768
7.762
7.722
7.673
7.663
7.659
7.650
7.568
7.560
7.556
7.551
7.544
7.536
7.502
7.497
7.479
7.475
7.453
7.446
7.438
7.436
7.431
7.423
7.411
7.257
7.241
7.236
7.228
8.8
8.6
8.4
8.2
8.0
7.8
7.6
7.4
ppm
20.10
10.12
19.62
10.57
31.10
67.52
30.00

YCY-5832

Pulse Sequence: s2pul
UNITYplus-400 "unity400"
Date: Jul 2 2018
Solvent: DMSO
Ambient temperature
Total 6304 repetitions

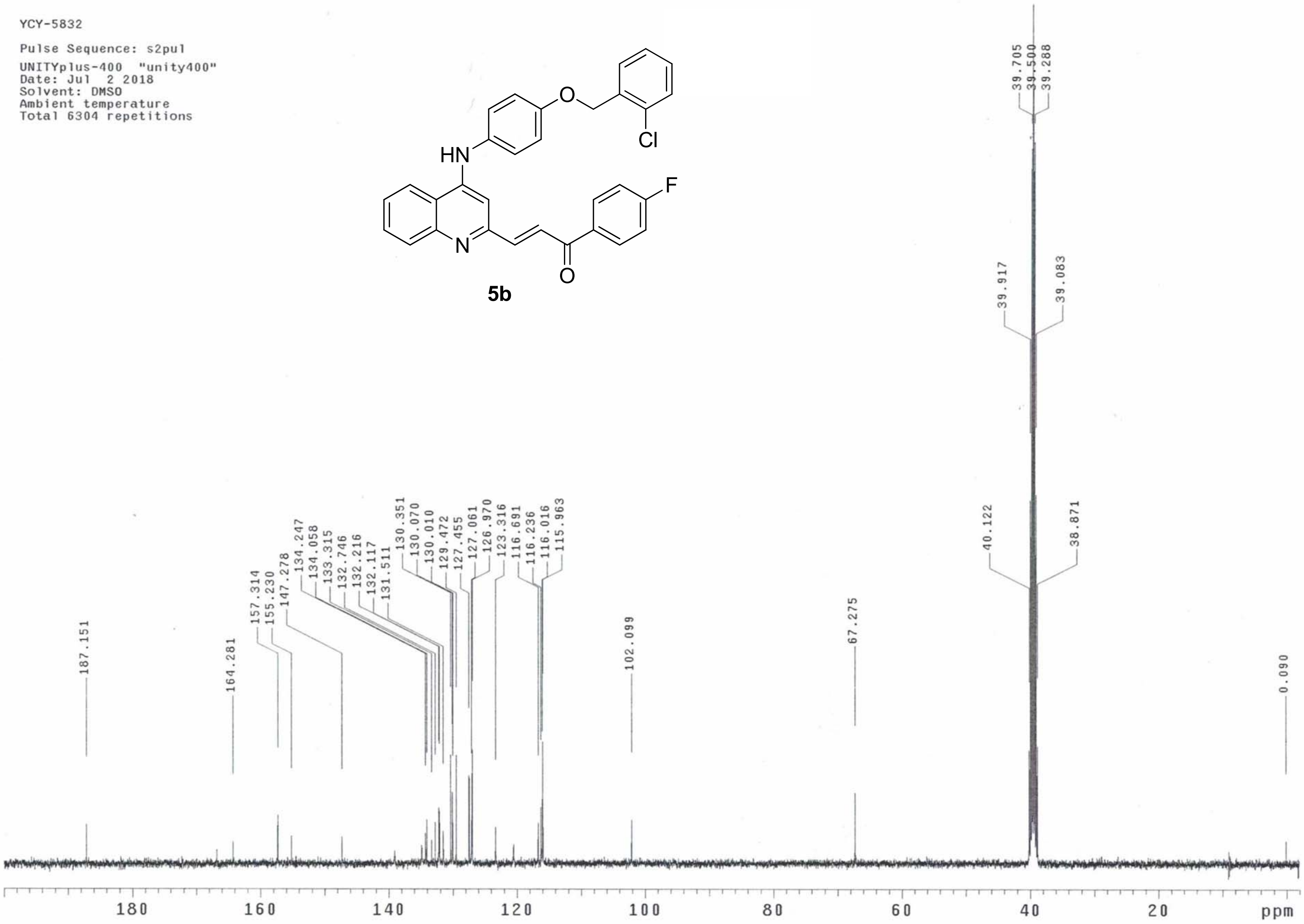

YCY-5836

Pulse Sequence: s2pul
Mercury-400BB "MerPlus400"
Date: Aug 16 2018
Solvent: dmso
Ambient temperature
Total 32 repetitions

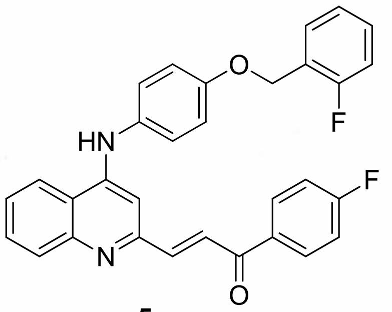

5c

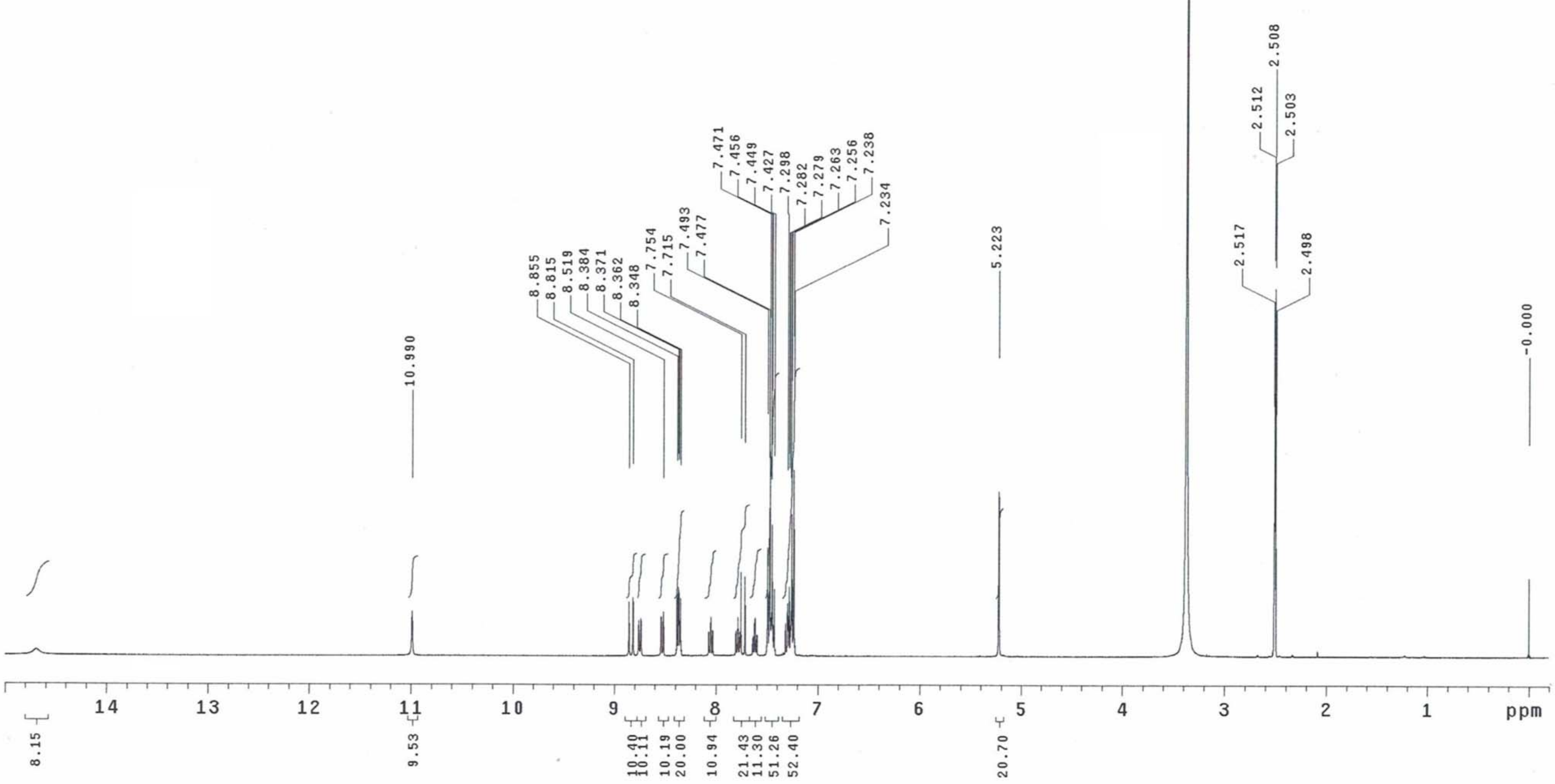

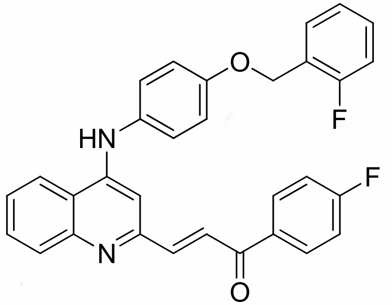
HN
O
F
F
N
O

5c

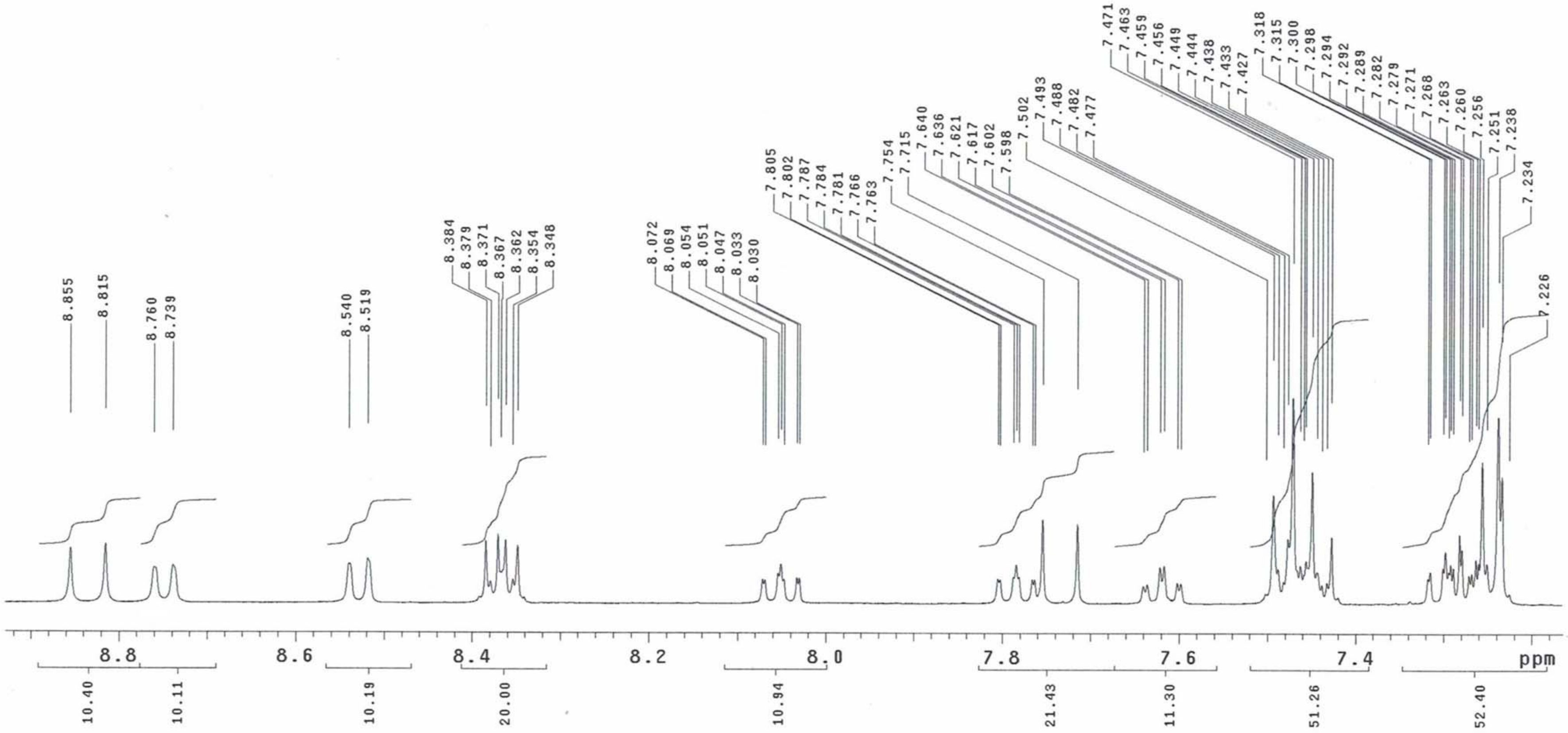
8.855
8.815
8.760
8.739
8.540
8.519
8.384
8.379
8.371
8.367
8.362
8.354
8.348
8.072
8.069
8.054
8.051
8.047
8.033
8.030
7.805
7.802
7.787
7.784
7.781
7.766
7.763
7.754
7.715
7.640
7.636
7.621
7.617
7.602
7.598
7.502
7.493
7.488
7.482
7.477
7.471
7.463
7.459
7.456
7.449
7.444
7.438
7.433
7.427
7.318
7.315
7.300
7.298
7.294
7.292
7.289
7.282
7.279
7.271
7.268
7.263
7.260
7.256
7.251
7.238
7.234
7.226
8.8
8.6
8.4
8.2
8.0
7.8
7.6
7.4
ppm
10.40
10.11
10.19
20.00
10.94
21.43
11.30
51.26
52.40

YCY-5836

Pulse Sequence: s2pul
Mercury-400BB "MerPlus400"
Date: Aug 16 2018
Solvent: dmso
Ambient temperature
Total 4752 repetitions

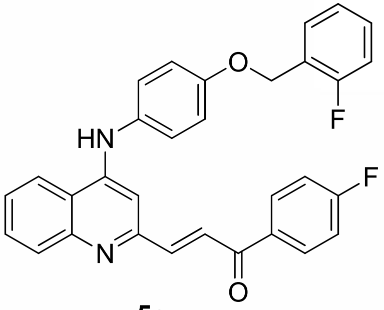

5c

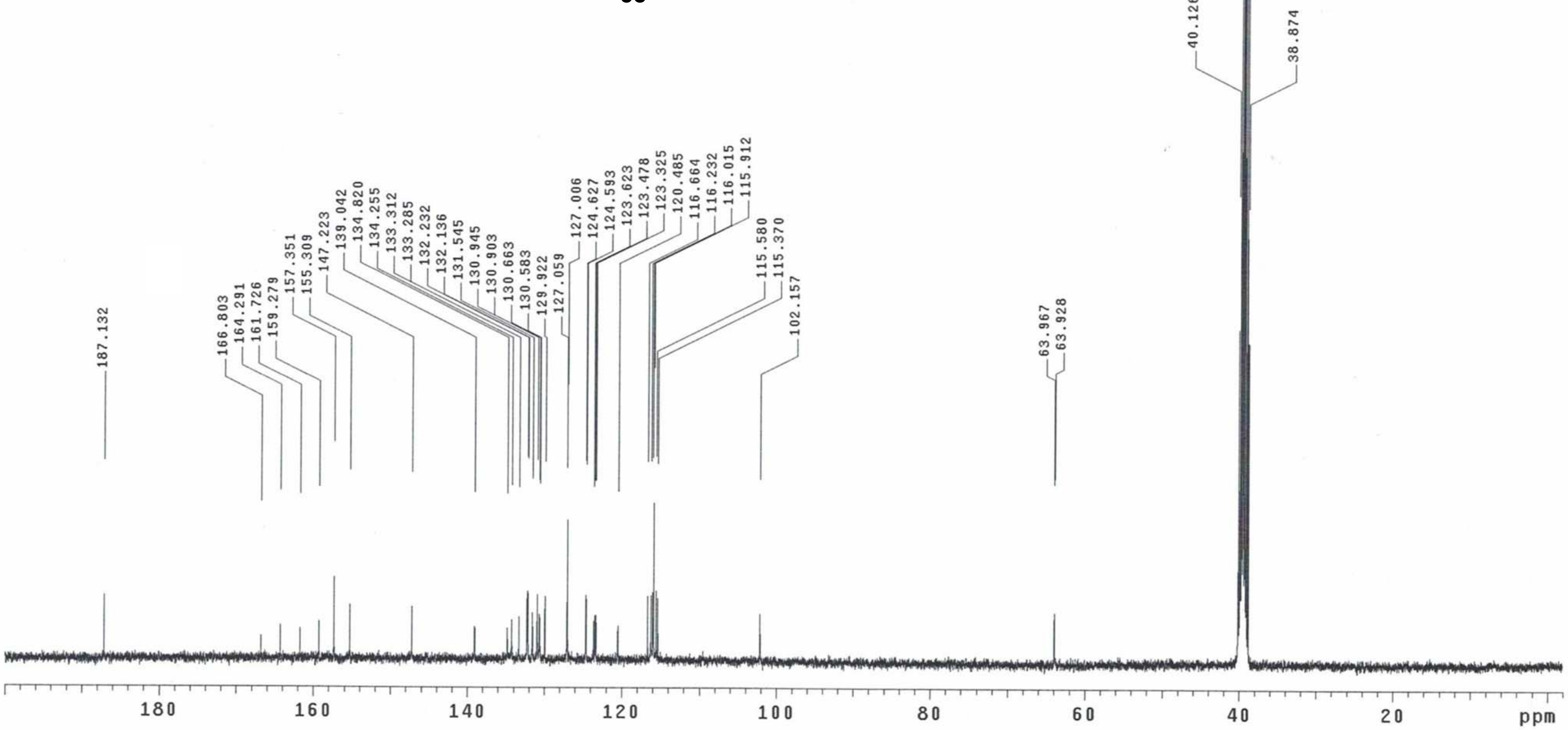

YCY-5841

Pulse Sequence: s2pul
Mercury-400BB "MerPlus400"
Date: Oct 5 2018
Solvent: dmso
Ambient temperature
Total 32 repetitions

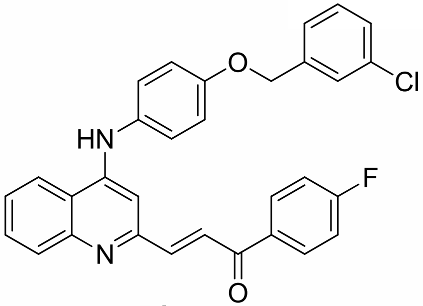

5d

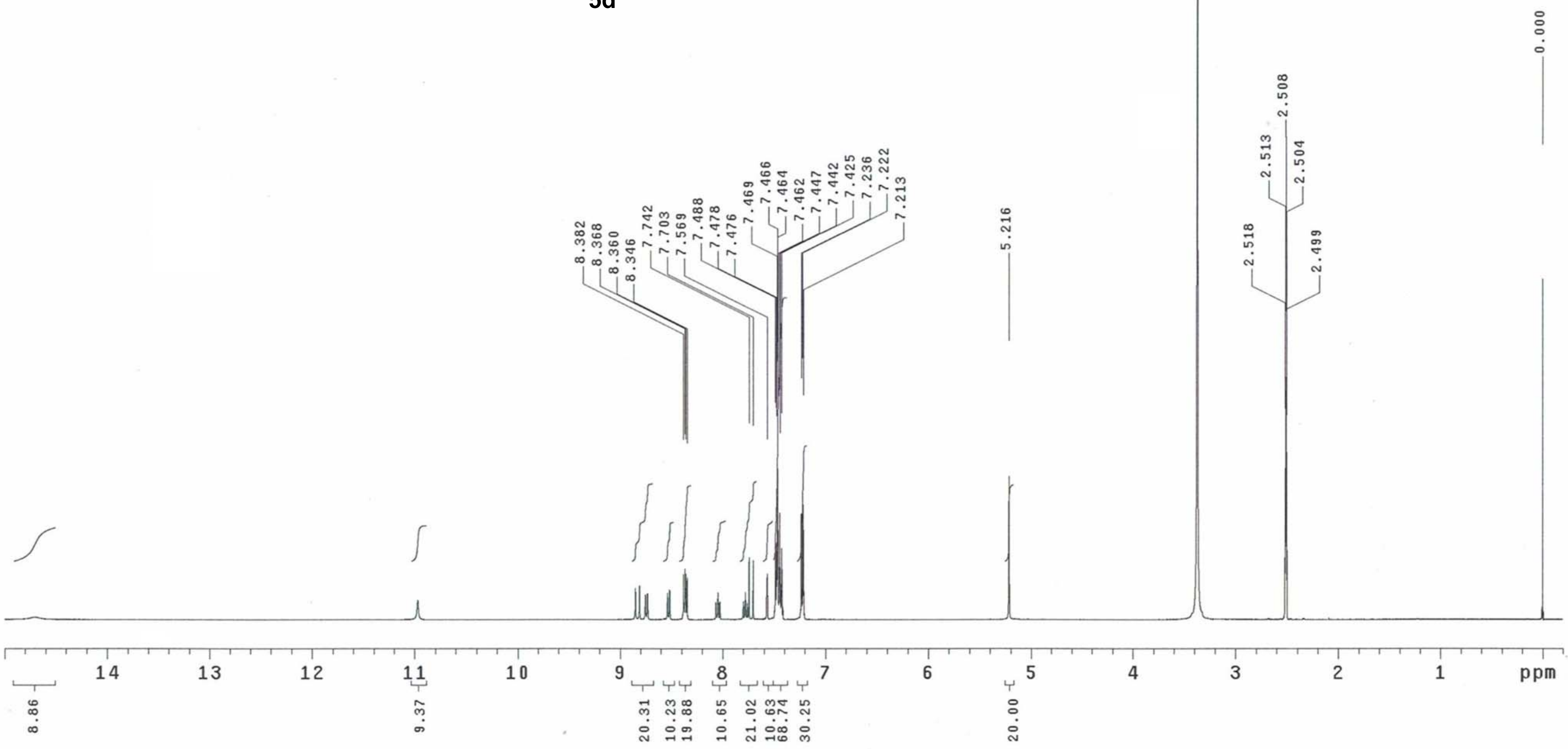

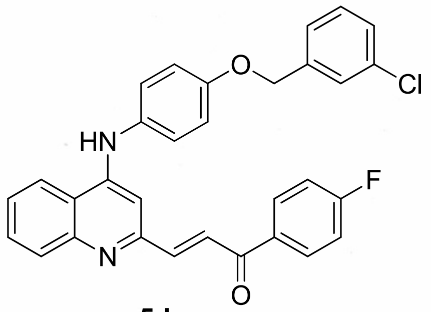

5d

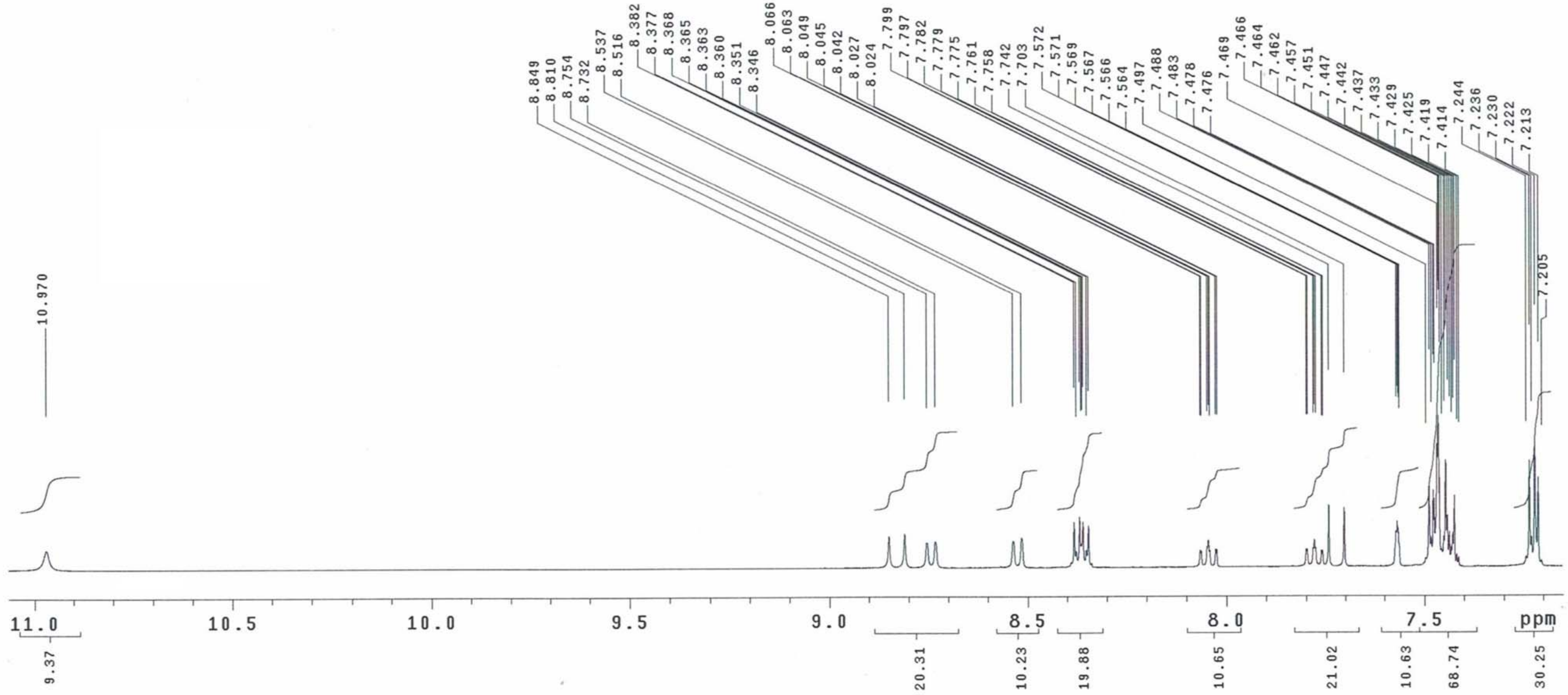

YCY-5841

Pulse Sequence: s2pul
Mercury-400BB "MerPlus400"
Date: Oct 5 2018
Solvent: dmso
Ambient temperature
Total 6368 repetitions

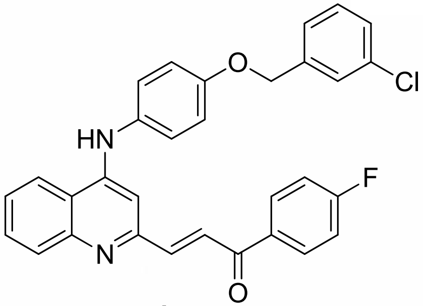

5d

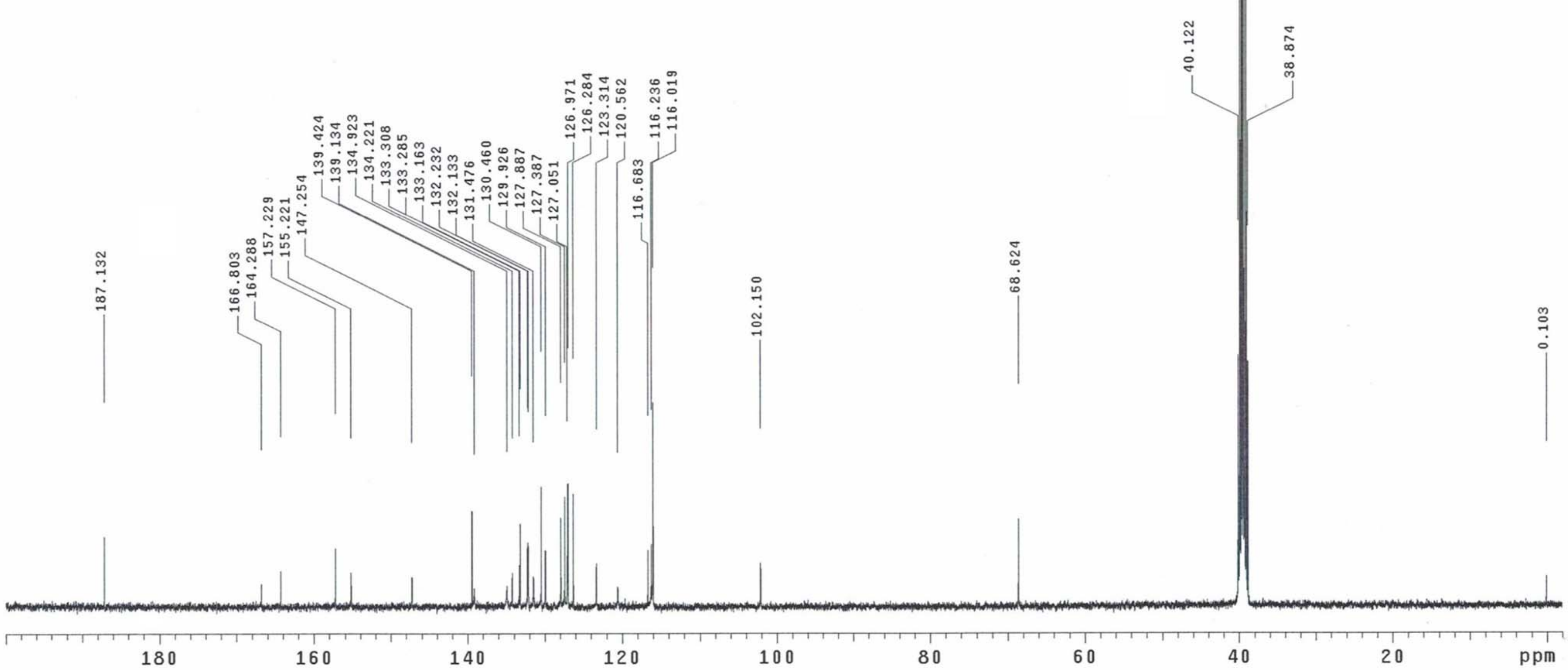

YCY-5837

Pulse Sequence: s2pul
UNITYplus-400 "unity400"
Date: Aug 16 2018
Solvent: DMSO
Ambient temperature
Total 64 repetitions

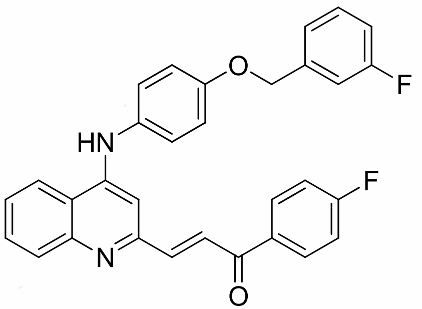

5e

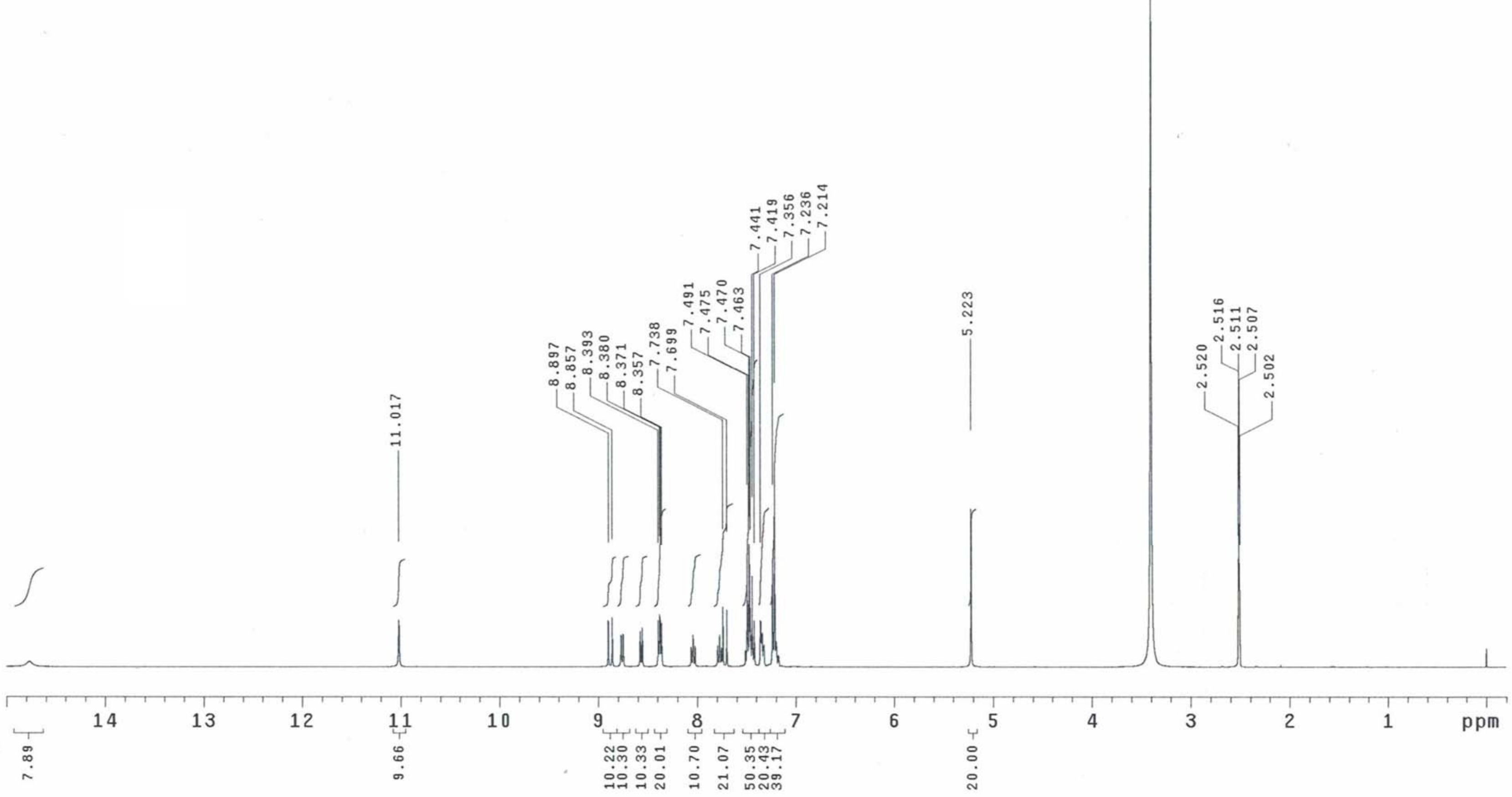

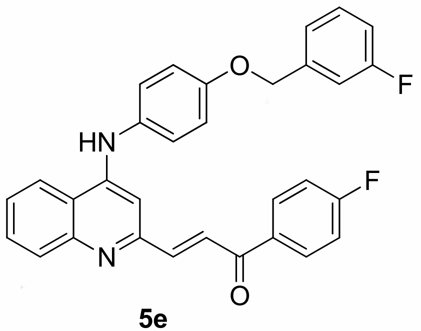
HN
O
F
F
N
O

5e

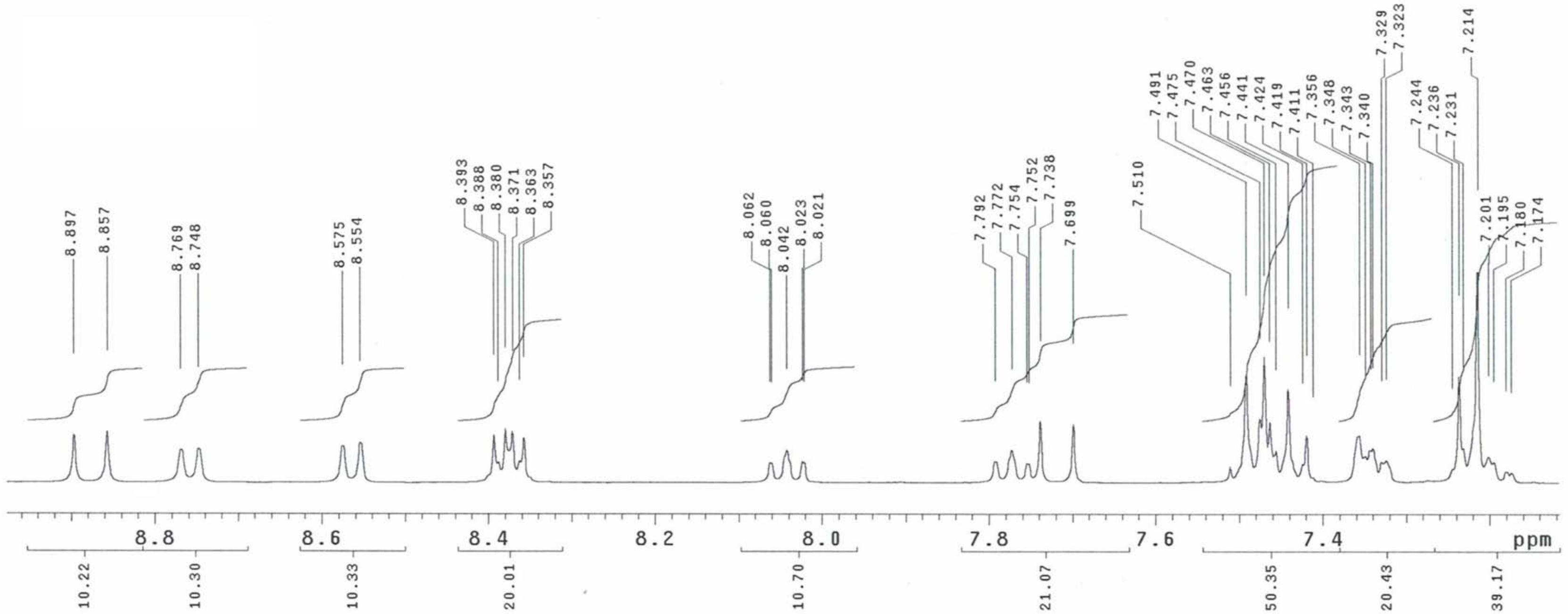
8.897
8.857
8.769
8.748
8.575
8.554
8.393
8.388
8.380
8.371
8.363
8.357
8.062
8.060
8.042
8.023
8.021
7.792
7.772
7.754
7.752
7.738
7.699
7.510
7.491
7.475
7.470
7.463
7.456
7.441
7.424
7.419
7.411
7.356
7.348
7.343
7.340
7.329
7.323
7.244
7.236
7.231
7.214
7.201
7.195
7.180
7.174
8.8
8.6
8.4
8.2
8.0
7.8
7.6
7.4
ppm
10.22
10.30
10.33
20.01
10.70
21.07
50.35
20.43
39.17

YCY-5837

Pulse Sequence: s2pul
UNITYplus-400 "unity400"
Date: Aug 16 2018
Solvent: DMSO
Ambient temperature
Total 16000 repetitions

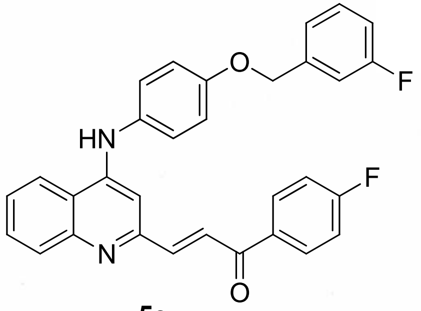

5e

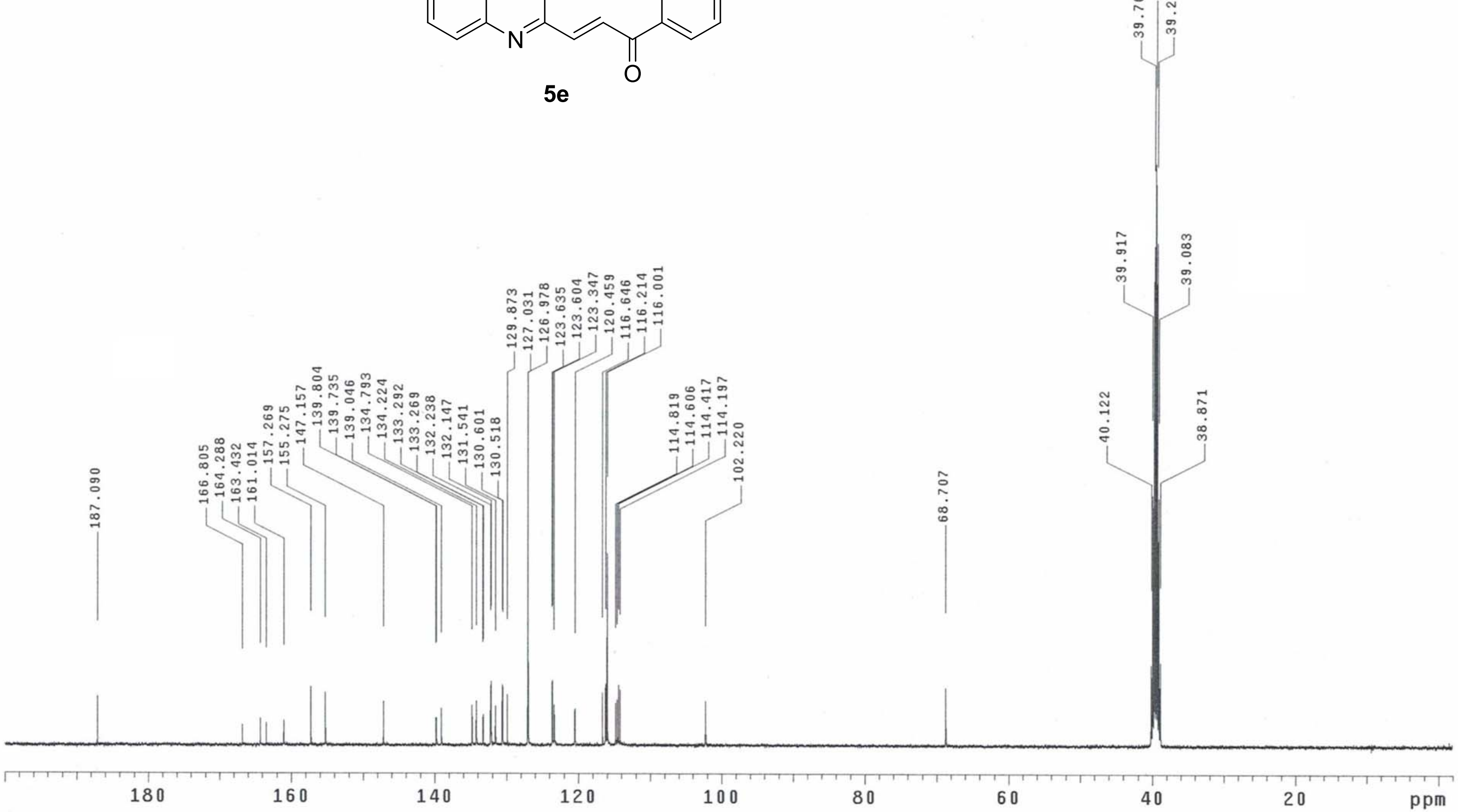

YCY-5838

Pulse Sequence: s2pul
UNITYplus-400 "unity400"
Date: Aug 16 2018
Solvent: DMSO
Ambient temperature
Total 64 repetitions

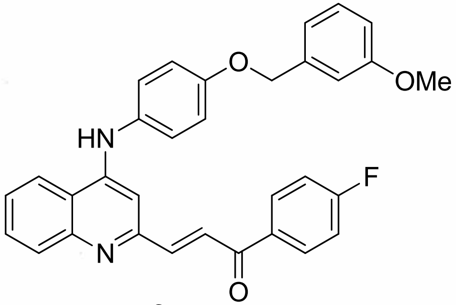

5f

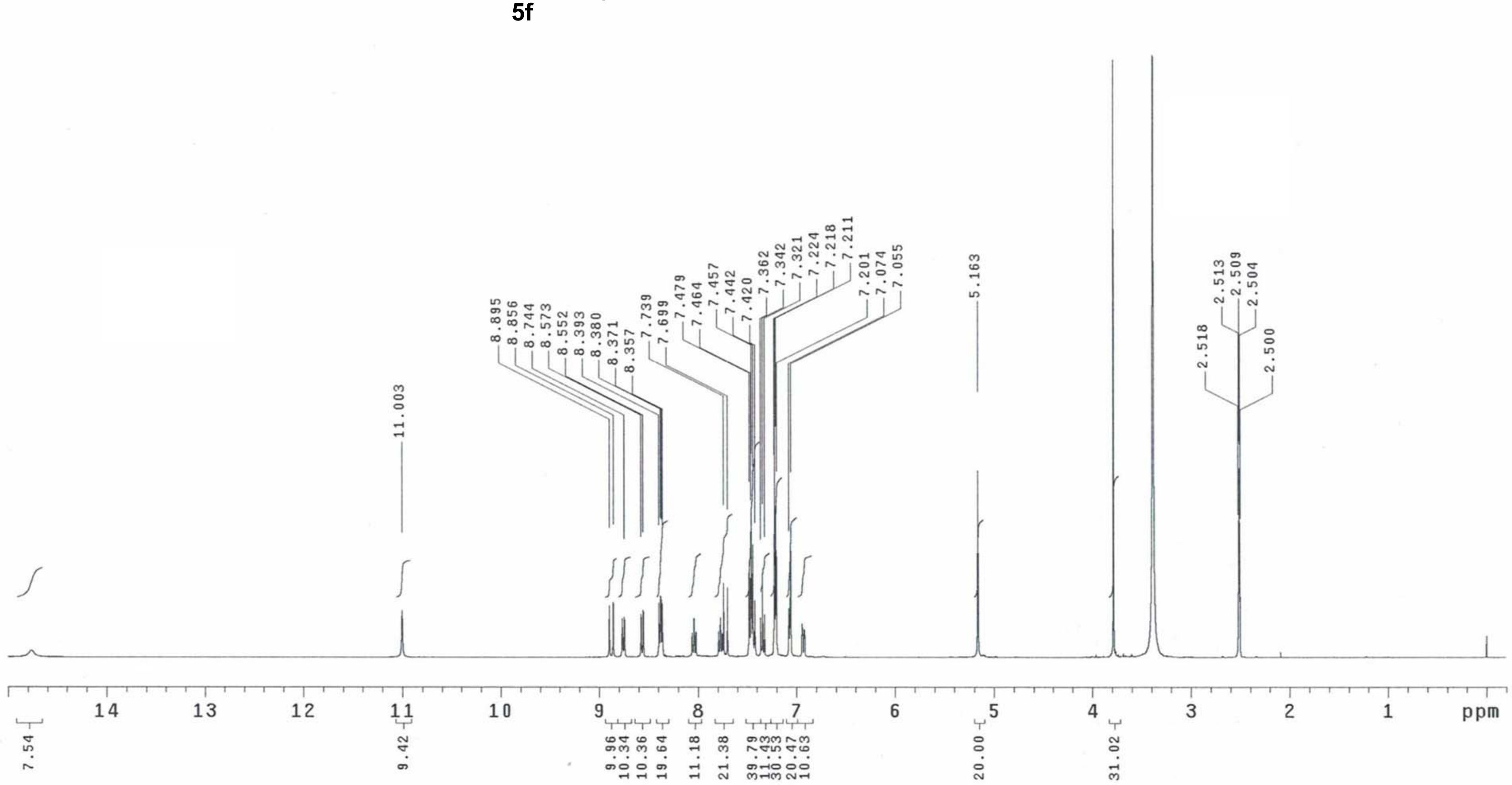

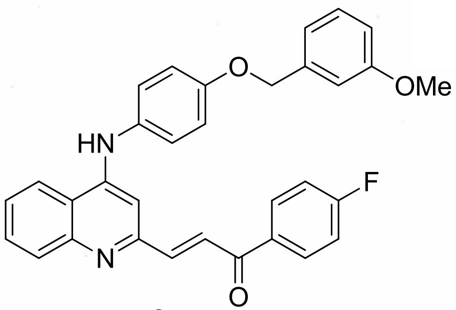

5f

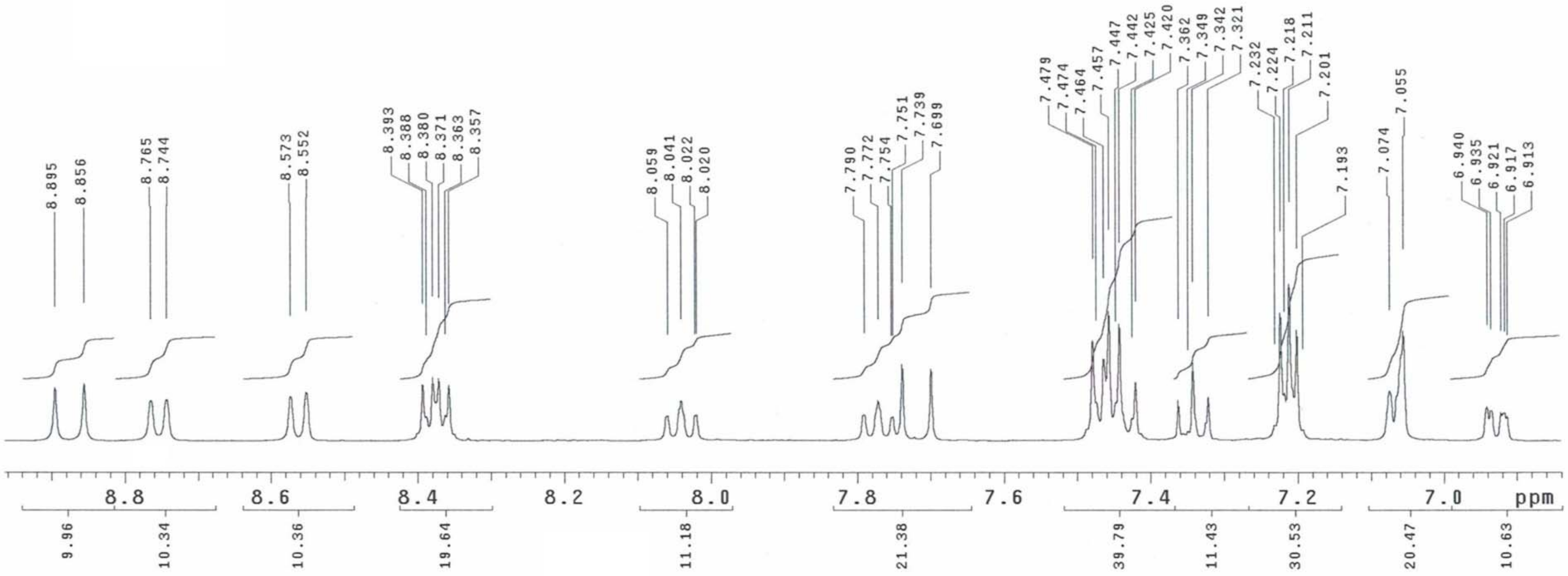

8.895
8.856
8.765
8.744
8.573
8.552
8.393
8.388
8.380
8.371
8.363
8.357
8.059
8.041
8.022
8.020
7.790
7.772
7.754
7.751
7.739
7.699
7.479
7.474
7.464
7.457
7.447
7.442
7.425
7.420
7.362
7.349
7.342
7.321
7.232
7.224
7.218
7.211
7.201
7.193
7.074
7.055
6.940
6.935
6.921
6.917
6.913
8.8
8.6
8.4
8.2
8.0
7.8
7.6
7.4
7.2
7.0
ppm
9.96
10.34
10.36
19.64
11.18
21.38
39.79
11.43
30.53
20.47
10.63

YCY-5838

Pulse Sequence: s2pul
UNITYplus-400 "unity400"
Date: Aug 16 2018
Solvent: DMSO
Ambient temperature
Total 3040 repetitions

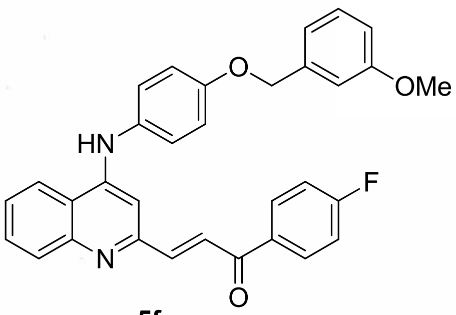

5f

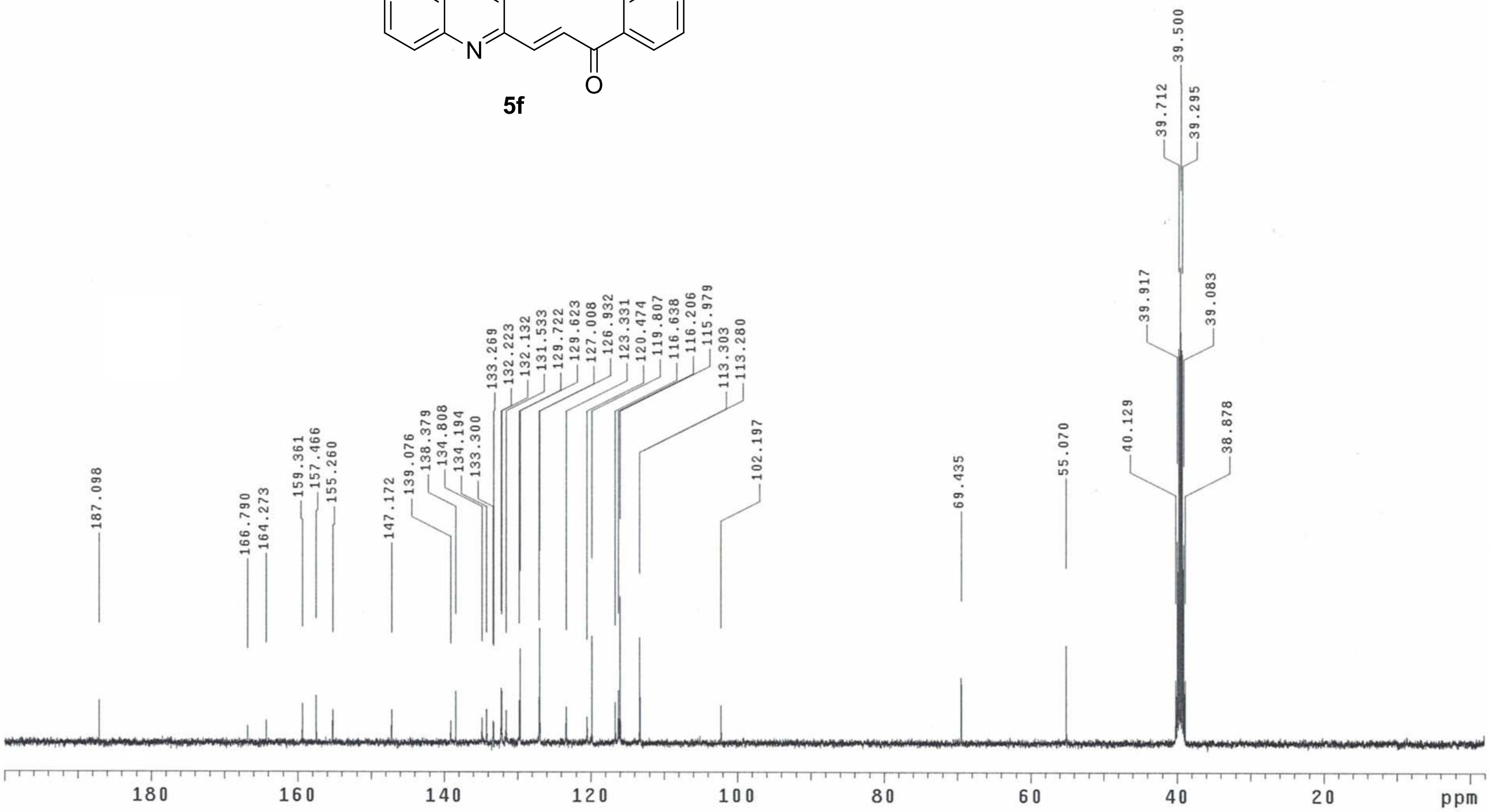

YCY-5833

Pulse Sequence: s2pul

UNITYplus-400 "unity400"
Date: Jul 4 2018
Solvent: DMSO
Ambient temperature
Total 32 repetitions

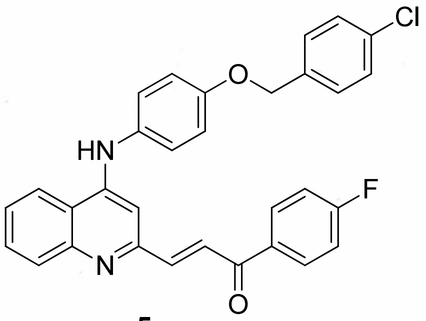

5g

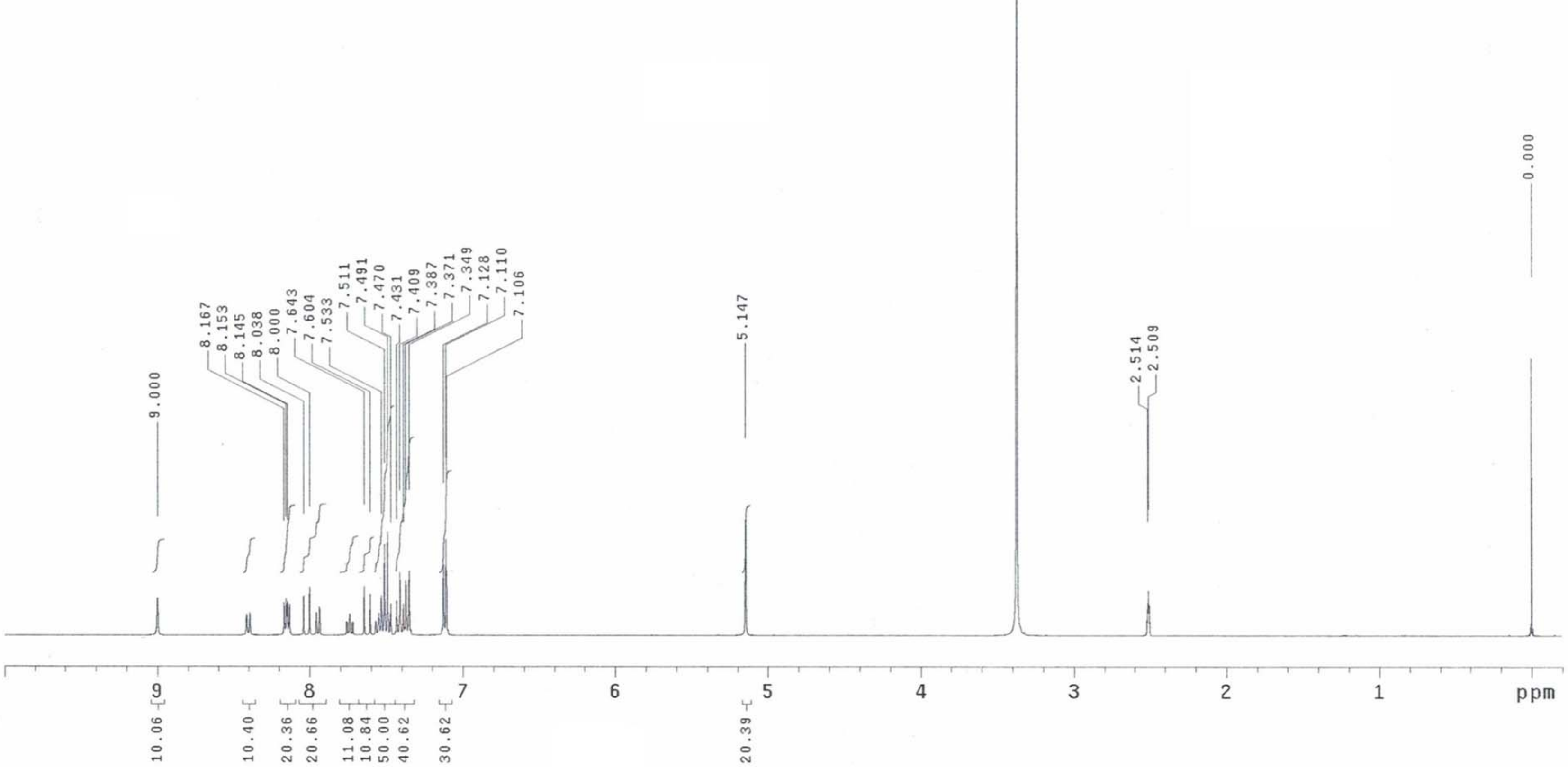

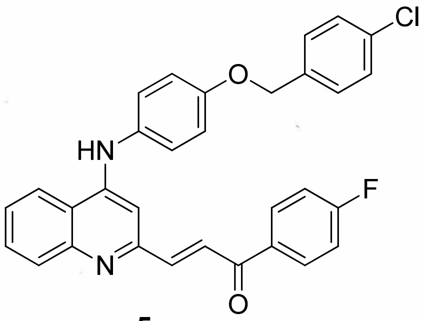
Cl
O
HN
N
F
O

5g

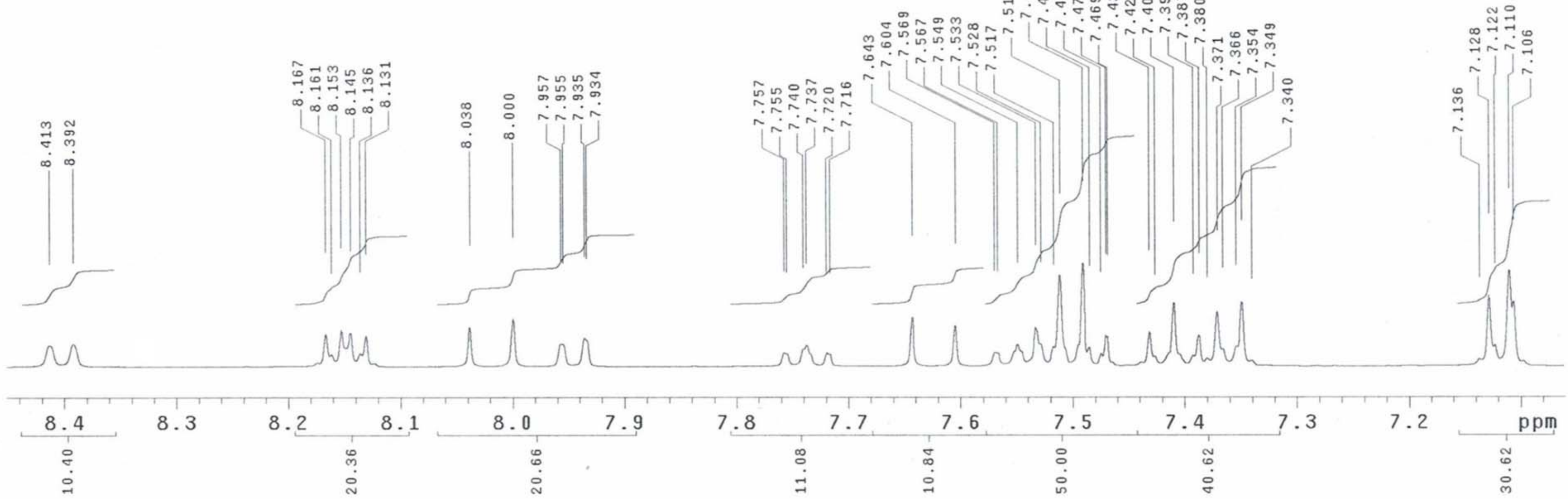
8.413
8.392
8.167
8.161
8.153
8.145
8.136
8.131
8.038
8.000
7.957
7.955
7.935
7.934
7.757
7.755
7.740
7.737
7.720
7.716
7.643
7.604
7.569
7.567
7.549
7.533
7.528
7.517
7.511
7.491
7.485
7.475
7.470
7.469
7.431
7.426
7.409
7.392
7.387
7.380
7.371
7.366
7.354
7.349
7.340
7.136
7.128
7.122
7.110
7.106
8.4
8.3
8.2
8.1
8.0
7.9
7.8
7.7
7.6
7.5
7.4
7.3
7.2
ppm
10.40
20.36
20.66
11.08
10.84
50.00
40.62
30.62

YCY-5833

Pulse Sequence: s2pul

UNITYplus-400 "unity400"
Date: Jul 4 2018
Solvent: DMSO
Ambient temperature
Total 3360 repetitions

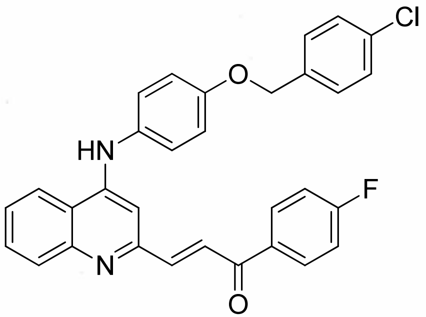

5g

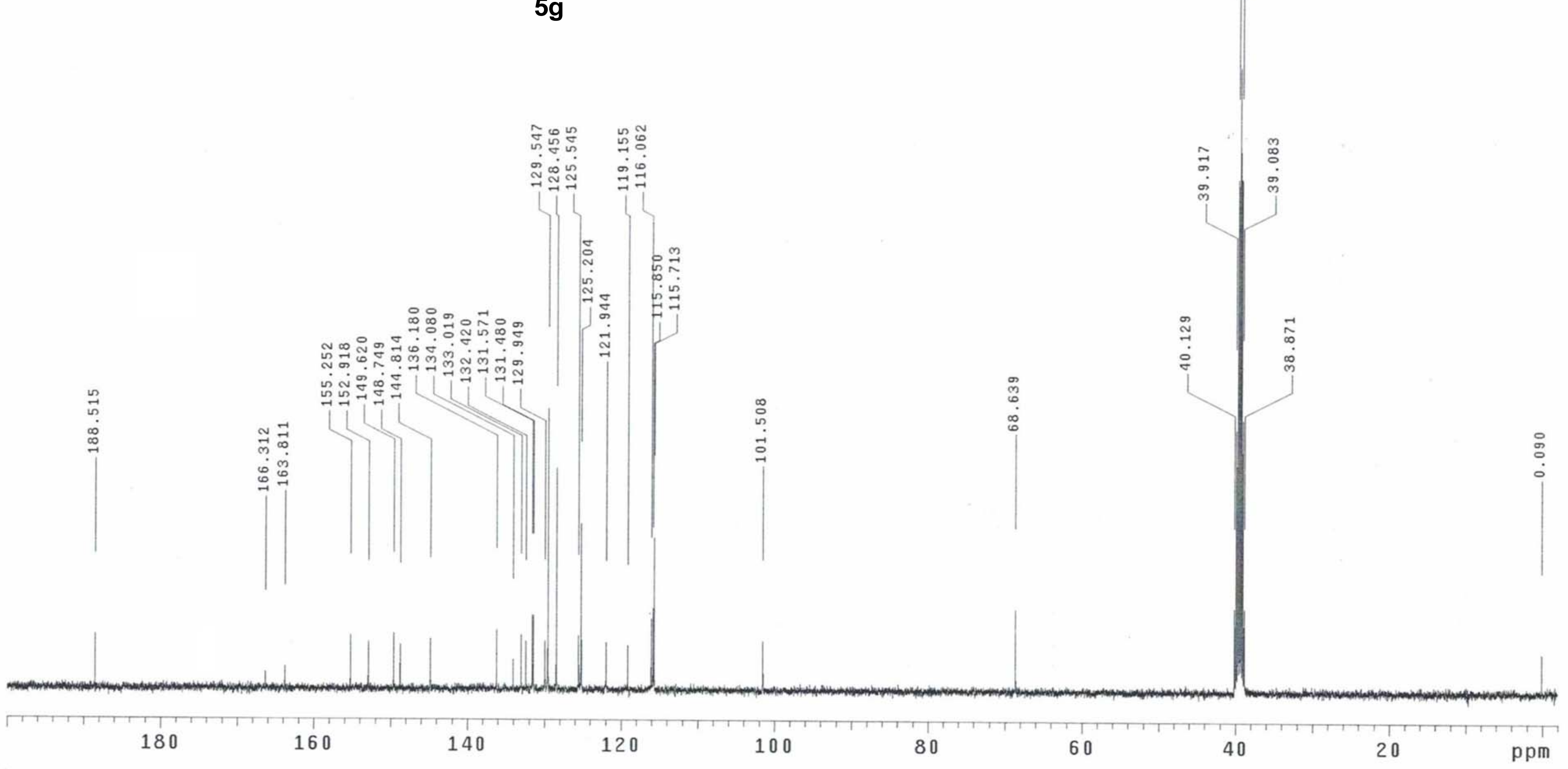

YCY-5840

Pulse Sequence: s2pul
Mercury-400BB "MerPlus400"
Date: Oct 5 2018
Solvent: dmso
Ambient temperature
Total 32 repetitions

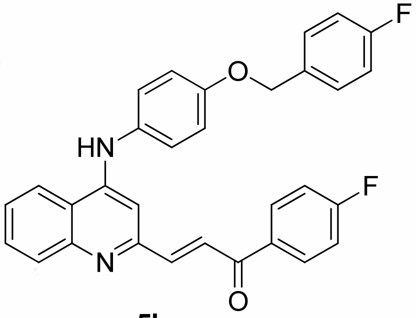

5h

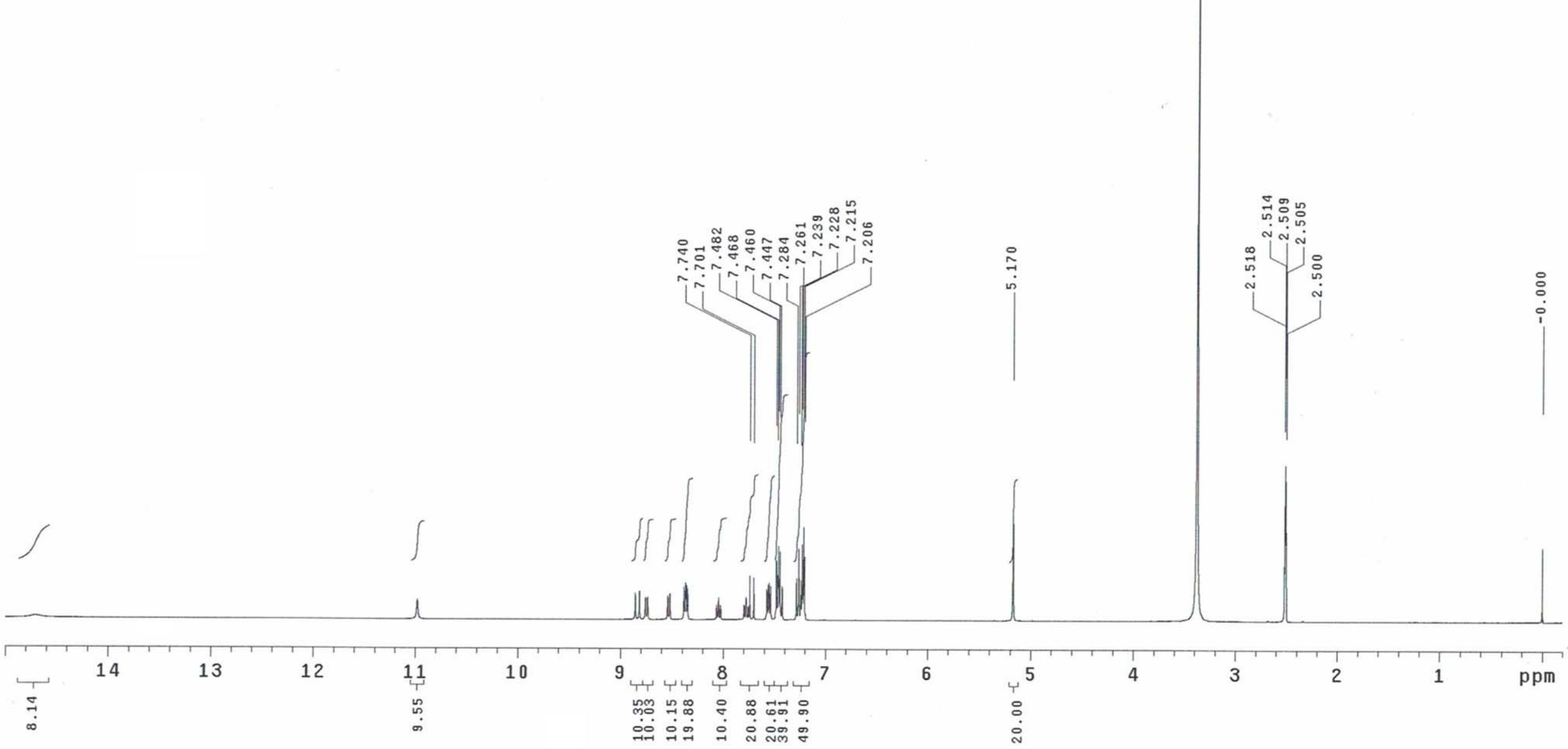

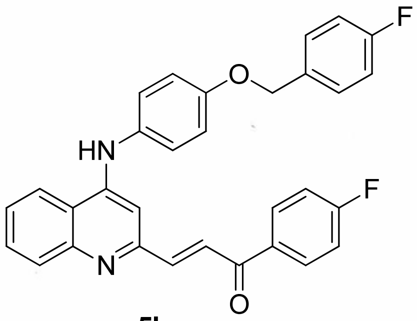

5h

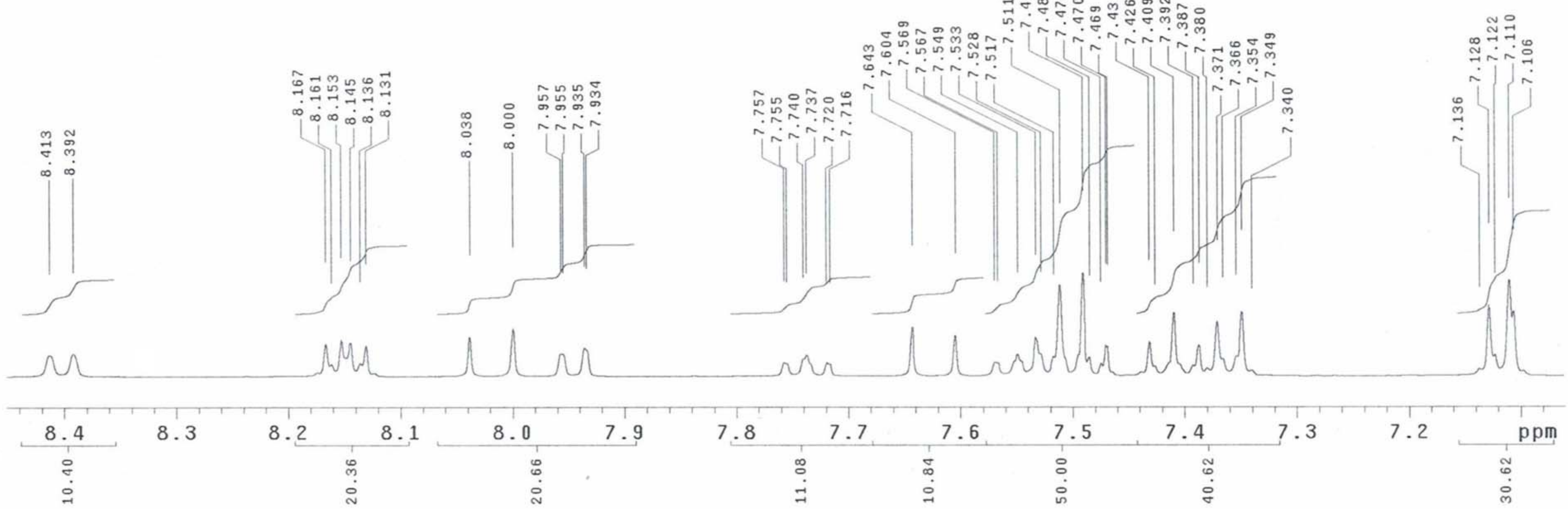
8.413
8.392
8.167
8.161
8.153
8.145
8.136
8.131
8.038
8.000
7.957
7.955
7.935
7.934
7.757
7.755
7.740
7.737
7.720
7.716
7.643
7.604
7.569
7.567
7.549
7.533
7.528
7.517
7.511
7.491
7.485
7.475
7.470
7.469
7.431
7.426
7.409
7.392
7.387
7.380
7.371
7.366
7.354
7.349
7.340
7.136
7.128
7.122
7.110
7.106
8.4 8.3 8.2 8.1 8.0 7.9 7.8 7.7 7.6 7.5 7.4 7.3 7.2 ppm
10.40
20.36
20.66
11.08
10.84
50.00
40.62
30.62

YCY-5840

Pulse Sequence: s2pul
Mercury-400BB "MerPlus400"
Date: Oct 5 2018
Solvent: dmso
Ambient temperature
Total 4880 repetitions

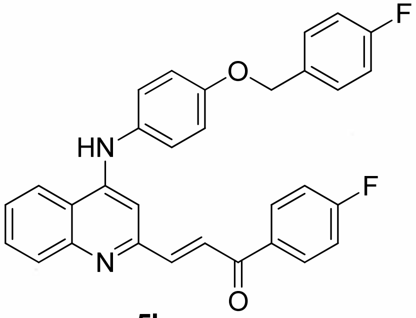

5h

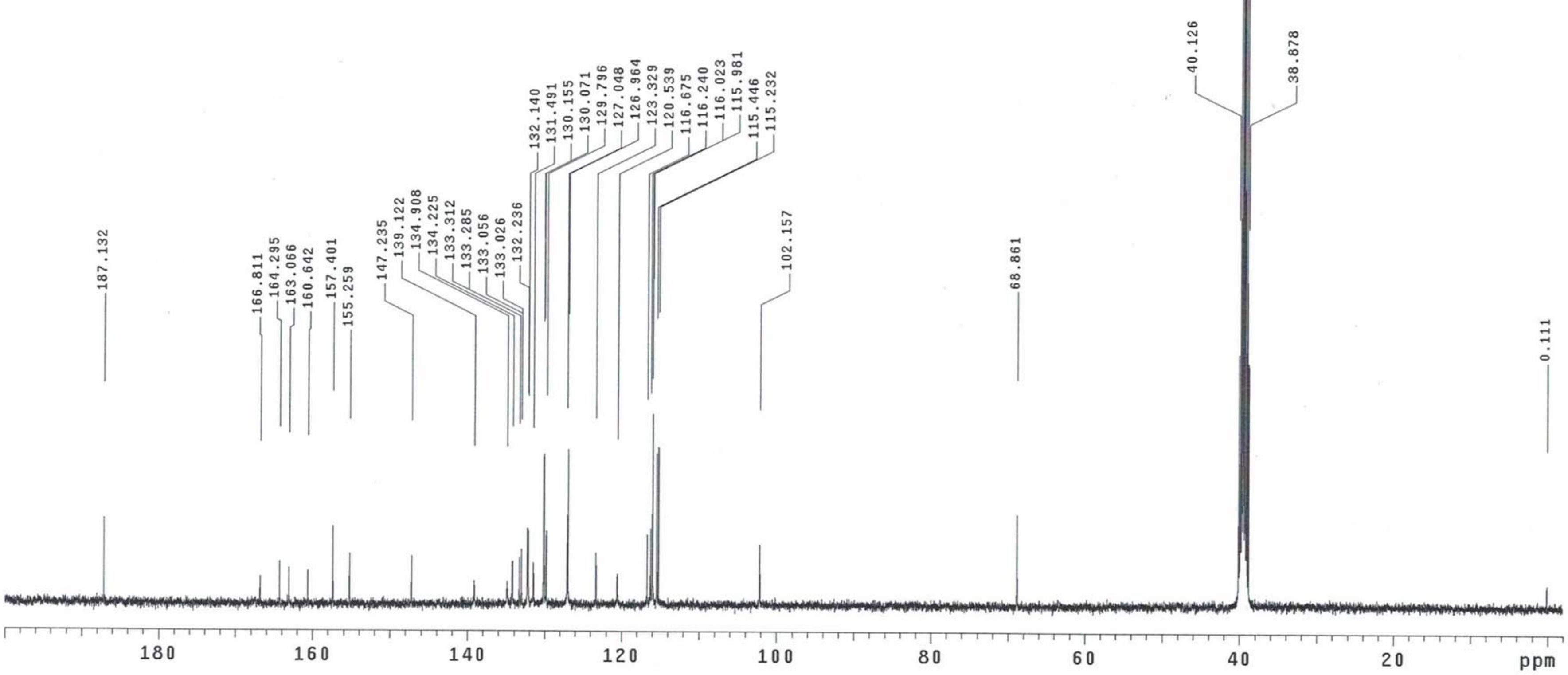

Supplement: Supplementary file 1 [file ijms-24-06034-s001.zip › ijms-2287274-supplementary.pdf]
